# A century of decoupling size and structure of urban spaces in the United States

**DOI:** 10.1038/s43247-020-00082-7

**Published:** 2021-01-27

**Authors:** Johannes H. Uhl, Dylan S. Connor, Stefan Leyk, Anna E. Braswell

**Affiliations:** 1 Department of Geography, University of Colorado Boulder, Boulder, CO, USA.; 2 School of Geographical Sciences & Urban Planning, Arizona State University, Tempe, AZ, USA.; 3 Earth Lab, Cooperative Institute for Research in Environmental Sciences (CIRES), University of Colorado Boulder, Boulder, CO, USA.; 4 University of Colorado Population Center (CUPC), Institute of Behavioral Science (IBS), University of Colorado Boulder, Boulder, CO, USA.

## Abstract

Most cities in the United States of America are thought to have followed similar development trajectories to evolve into their present form. However, data on spatial development of cities are limited prior to 1970. Here we leverage a compilation of high-resolution spatial land use and building data to examine the evolving size and form (shape and structure) of US metropolitan areas since the early twentieth century. Our analysis of building patterns over 100 years reveals strong regularities in the development of the size and density of cities and their surroundings, regardless of timing or location of development. At the same time, we find that trajectories regarding shape and structure are harder to codify and more complex. We conclude that these discrepant developments of urban size- and form-related characteristics are driven, in part, by the long-term decoupling of these two sets of attributes over time.

By 2050, more than two thirds of humans will live in urban areas^1^, a striking projection considering that the estimated share of people living in cities was below 30% in 1950 and less than 16% in 1900^2^. While we do not know what form cities will take in the future, we can be certain that their organization and structure will increasingly influence climate change, human health, economic development and social inequality^3-6^. Unfortunately, our efforts to envision the cities of the future are constrained by the absence of systematic insight on how today’s cities and their surroundings have changed in the past. Much of this shortfall is attributed to the absence of detailed information on urban spatial change prior to the 1970s.

Historical analysis and theory provides conflicting perspectives on long-term urbanization. While technological change is enabling the creation of increasingly complex cities^7^, debate continues as to whether differences across urban areas are decreasing or if regions are retaining distinctive and enduring properties^8,9^. This debate has long occupied historical scholars, who are increasingly skeptical of the notion that universal forces are homogenizing our progressively urban world^10^. The homogenization of cities is, therefore, a continuing discussion point for geographers, social theorists and historians.

Urban spatial data are enabling investigation into urban development, including the examination of the bottom-up selforganization of city systems, how the characteristics of cities scale with growth, and how different urban attributes change to create related and potentially predictable patterns (“allometry”)^11,12^. These scaling and allometric relationships, in terms of urban size, density, and form^13-15^ are highly consequential for a wide range of socio-economic and environmental outcomes^16-18^. Unfortunately, the paucity of long-term urban spatial data generally confined studies to relatively short time horizons or selected geographic contexts^19,20^, making it difficult to fully understand urban growth, or (changes in) urban form^21-24^ and similarity^25-27^. As such, much of our knowledge of urban change rests on cross-sectional data and relatively short windows of observation that do not fully encompass the complete development trajectories of cities.

Our objective is to shed new light on the question of *how the spatial characteristics of cities and their surroundings have changed over the last century*. In answering this question, our goal is to determine the consistency and variance of long-term urban spatial development with respect to city size, density, shape and structure. We do so by leveraging our recently published Historical Settlement Data Compilation for the US (HISDAC-US)^28-31^ derived from the Zillow Transaction and Assessment dataset (ZTRAX)^32^ to examine urban spatial development within *contemporary* metropolitan statistical areas (hereafter “MSAs”) in terms of size, density, shape, and structure related metrics^33,34^. ZTRAX is a compilation of land use, built, valuation and real-estate characteristics for over 200 million US cadastral parcels, and contains rarely examined building attributes, including the year built, indoor area, total housing units, and structural characteristics (e.g., building material, function). We contend that the gridded spatial data layers from HISDAC-US are currently the most compelling source of high-resolution data on the long-term spatial development of the United States.

We use HISDAC-US to observe spatial land use changes within the boundaries of MSAs, as defined in 2010. As MSA boundaries delineate the proximate socio-economic influence of urban centers (see Methods section), they provide the most appropriate spatial units for understanding the emergence of the contemporary US urban system. We examine spatiotemporal land use changes for MSAs using our compilation of settlement data (Fig. 1) to derive time series of descriptive spatial variables for each MSA in the US at semi-decadal temporal resolution from 1910 to 2010. Using 10 metrics inspired by commonly employed landscape metrics^35-37^ to characterize urban areas (Table 1; see Methods section for detailed explanations), we utilize descriptive visualizations, statistical methods and cluster analysis to identify common types of urban spatial development over the last century These metrics are categorized into size-related and form-related (i.e., shape and structure) characteristics that can be used to analyze the interactions between these attributes^11^.

Our analysis proceeds in four steps. First, we examine nationwide and MSA-specific development trends among the time series of our 10 urban-spatial metrics. After describing these development paths, we perform cluster analysis on temporal cross-sections of our data to analyze historical changes in the spatial properties of MSAs and their movement between different clusters over time. Third, to understand variations in urban development, we decompose these trends spatially for different regions. We conclude our analysis with an ordinal assessment of the relationships between size and form related urban-spatial metrics, in order to investigate whether our numerical analysis findings also hold in an ordinal scale. In this study, we will refer to the term “MSA” as the analytical unit used but will use both “MSAs” and “cities” interchangeably in discussing and interpreting results.

From this analysis, we provide evidence of a weakening association between the size-related attributes of MSAs with their respective form-related characteristics since the early twentieth century. That is, we find that the correlation between urban size and urban form in our data has declined substantially from 1910 to 2010. This attenuation appears to be driven by the growing similarity of places with respect to their size attributes over time, but a greater endurance of their form-related attributes across the same period. One potential limitation of our analysis is that the underlying ZTRAX data capture the *existing* building stock of the United States. Thus, we do not observe buildings that have been torn down or urban footprints that have shrunk^38,39^. The selective nature of replacement at the *building scale*^40,41^ could therefore bias our findings at the *metropolitan scale.* We find that our main results are highly robust, implying that survivorship bias is not likely to be driving our key results (see Methods section). Nonetheless, the proceeding analysis should be viewed as an examination of buildings that have survived from their initial built year to 2010.

## Results

### Nationwide development trends since 1910.

We begin by analyzing the trends in the size (Fig. 2a) and form metrics (Fig. 2b) across MSAs from 1910 to 2010. With four of the five size-related variables increasing over our study period, the size variables are almost exclusively lower in magnitude in 1910 than in 2010 across all MSAs. Thus, we observe nationwide increases in urban size (BUAREA, NUMBUPROP) and density (BUDENS, NETBUI) over the last century. While this pattern is expected due to the nature of our data, it is consistent with findings from global meta-analyses of city size changes^6^.

At the building scale, average housing unit size (AVGHUSIZE) follows a V-shape, being at similarly high levels in 1910 and 2010 with a minimum in 1950. This finding suggests on average similar levels of size among housing units existing in the United States in 1910 and 2010. The decline of housing unit size in the first half of the twentieth century implies increasing construction of multi-family homes and apartment buildings (i.e., containing relatively small housing units). The increase in AVGHUSIZE starting from the 1950s likely reflects the expansion of singlefamily home construction in the mid-twentieth century^42^. This pattern corresponds to the primary era of US suburbanization^43^, as depicted in Fig. 2, resulting in increasing proportions of housing units larger than those in apartment buildings. The trend is consistent with census-based findings^44^ but may be subject to survivorship bias in early settled areas (see Methods section).

The form variables vary more than the size-related characteristics. The spatial dispersion measures scatteredness (SCAT), number of patches (NUMPATCH), and the proportion of area accounted for by the largest single contiguous patch of urbanized land (MAXPATCHPROP) increased over the first 70 years of our record. This trend implies that both the number of settlements and their contiguous area increased over time. Since roughly 1970, the spatial dispersion measures flattened out and are now beginning to decline. This change reflects the transition from discrete areas of development around developing cities to more contiguous urban spatial environments or a “filling in” of contemporary urban regions over time.

Relatedly, spatial compactness measures such as clusteredness (CLUST) and circularity (CIRC) have sharply declined over the 1910 to 2010 period. Over the century of observation, this downward trend shows little sign of reversal. Combined with the findings above, this suggests that the areas occupied by contemporary MSAs tended to consist of numerous but spatially compact settlements in the past, and over time, these settlements blended into more dominant contiguous urban areas that are quite complex in their forms. In short, the new building of urban land within MSAs is strongly associated with the rise of more contiguous but spatially dispersed built-up areas. These general trends are, to large degrees, invariant to the spatial resolution of the underlying gridded data, as indicated by a sensitivity analysis (Methods section) reproducing these trends based on gridded surfaces at multiple resolutions, as Supplementary Fig. 1 shows. Moreover, we conducted a population-based sensitivity analysis of our results to the survivorship bias by using the ratio of population counts to buildings over time to calculate likely measurement error in the enumeration of historical buildings per MSA. This sensitivity analysis suggests that the trends reported in Fig. 2 are largely insensitive to the underreporting of old buildings and the survivorship bias discussed above (Methods section, Supplementary Fig. 2).

### Convergence and dispersion of MSA trajectories.

While the descriptive statistics reveal substantial change in the spatial structure of MSAs over time, they tell us little about how urban characteristics are shifting relative to one another. Hence, we use a dimensionality reduction approach (t-distributed stochastic neighbor embedding; t-SNE; see Methods section) to represent the development of each MSA as a trajectory from 1910 to 2010 within two-dimensional attribute spaces for size and form (Fig. 3). The size-related variables of MSAs follow smooth (mostly parallel) bundled trajectories over time (Fig. 3a). MSAs appear to be relatively heterogeneous in size in the early twentieth century, converging in the later decades of the time series. While some MSAs were well developed by 1910 (i.e., exhibiting high BUAREA and NUMBUPROP values, such as NYC and Chicago), others grew quickly over the 20th century. This distinction is evident when early-developing northern, industrial urban regions such as around Boston, New York, and Chicago that are similarly situated in 1910 and 2010 are compared to more recently developing MSAs (e.g., Phoenix and Dallas) that are highly dispersed across the attribute space in 1910.

Our results underscore a unidirectional convergence process that reflects the growing but staggered historical development of MSAs. The observed general trend is expected due to the nature of the underlying data (i.e., not capturing shrinkage). However, high levels of congruence between 2010 radar charts (Fig. 3), and, to a lesser degree, among 1910 and 2010 radar charts, indicate relatively stable relationships between size variables among MSAs of different size, and over time, respectively. While we observe this wider process of convergence among MSAs by size, we also note recent patterns of size-related differentiation. The data reveal the emergence of a small set of mega-regions (e.g., New York, Atlanta) over recent decades, which exhibit extreme levels of built-up intensity in 2010. These MSAs generate non-normality in the data distributions of our variables, which is reflected in time series of cross-sectional Shapiro-Wilk normality test scores, as shown in Supplementary Fig. 3.

With regard to the form-related variables, we find that contemporary MSAs have developed along a more dispersed set of trajectories over time (Fig. 3b). We observe that MSAs in early developing, already industrialized urban regions (e.g., New York City, Boston, San Francisco) move from left to right through the attribute space, driven by decreasing clusteredness, scatteredness and total number of patches. More recently developing MSAs (e.g., Atlanta, Kansas City), in contrast, tend to move toward the upper left part of the attribute space, driven by decreasing compactness attributes (i.e., circularity, clusteredness). While it may appear that historical and contemporary development patterns are distinctly different, there is evidence of strong continuities. Later developing MSAs, such as Kansas City and Atlanta (in 2010), and earlier-developing MSAs, such as New York and Boston (in 1910), are similarly situated in the attribute space. These MSAs may, therefore, exhibit similarity in their form-related development, but these trajectories appear to be more complex than for the size-related attributes.

These findings provide strong descriptive evidence that MSAs follow systematic development paths through time with respect to size, but to lesser degrees, for form-related variables. Moreover, regionally decomposed versions of these trajectory plots (Supplementary Figs. 4, 5) suggest regional differences in these development paths. Although there is evidence that the form-related development of MSAs also exhibits similarities over time, these trajectories are considerably more complex than for size-related attributes, as indicated by the standard deviations of trajectory azimuths between subsequent years (Supplementary Table 2).

### Trajectories of MSA types.

Using the temporal cross-sections of MSA-level variables, we test for common forms of urban spatial development. We use the BIRCH clustering algorithm (see Methods section) to assess whether MSAs followed common development paths or if, instead, they tend to sort into specific clusters of urban spatial development. We apply cluster analysis separately to each temporal cross-section (Fig. 4a-c) and report intra-cluster and inter-cluster distances to measure within-cluster variance, and between-cluster separation, respectively (Fig. 4d, e). Moreover, we detail the variables underlying each cluster center (i.e., the medoid MSA in the attribute space), and visualize the geographic cluster distributions (Fig. 5). To facilitate interpretability, we focus on the top three variables, ranked by variance explained (i.e., principal component analysis factor loadings, see Methods section, Supplementary Table 1).

Based on the changes in clustering and the size of clusters (Fig. 4a, b), we can make several broad observations regarding the grouping of MSAs. First, for both categories, the number of clusters increases between 1910 and 2010. This finding implies that MSAs are separating into an increasing number of delineable spatial forms over time. The stability of within-cluster variance and increasing levels of cluster separation (Fig. 4d, e) are consistent with this interpretation. This result suggests that as MSAs scale up (i.e., grow), they become more highly stratified and increase in complexity (as indicated by the number of clusters).

The clusters based on the size-related variables (Fig. 4a) complement the interpretation of the prior analysis results. In 1910, the areas occupied by contemporary MSAs can be categorized into two types: built-up and sparsely settled areas. This interpretation is supported by clusters of MSAs with moderate numbers of built up properties (NUMBUPROP) and built-up intensity (NETBUI) and low levels of overall built up area (BUAREA) in 1910 (Fig. 5c). Since the mid-twentieth century, we observe that these two initial clusters split into four groups that generally persist up to 2010. This split reflects the growing stratification of MSAs over the twentieth century in terms of size. By 2010, we find four size-related MSA clusters that can be distinguished mostly by their built-up area (BUAREA), with fewer MSAs contained in these clusters.

For each point in time, the size-related cluster centers are almost perfectly nested inside one another (Fig. 5a-c). This finding suggests that while contemporary MSAs certainly differ with respect to size, the fundamental relations between size-related characteristics remain quite consistent through time. This regularity suggests that MSAs exhibit high levels of size-related stratification rather than strongly heterogeneous size-related spatial development. However, as mentioned before, it is important to note that due to the nature of the data used herein, the shrinkage of built-up area cannot be measured, and thus, our observations do not account for land conversion from built-up to not built-up (see Methods section).

By contrast, we observe more complex differentiation in the form of cities over time. There are five clusters in 1910 and seven clusters in 2010 (Fig. 4b). While clusters are mostly driven by differences in attribute magnitudes (as in the case of the size-related clusters), we also observe new and distinct cluster types between 1960 and 2010. Specifically, we find growing distinctiveness based on the contiguity measure MAXPATCHPROP. While the largest cluster of urban regions in 2010 (black) is associated with moderate MAXPATCHPROP, the second largest cluster (blue) captures MSAs with a high proportion of area covered by contiguous built-up land (e.g., high MAXPATCHPROP values, see also Fig. 5g). Thus, in addition to changes in the general magnitude of attribute values, the main distinction between the two largest form clusters is whether or not MSAs are characterized by large contiguous areas of built-up land.

To assess potential interactions and access the variability of attributes within clusters of urban regions across the two categories over time, we conducted several statistical analyses. We assessed the (univariate) distributions of each size variable within form-based clusters, and vice-versa, and found increasing levels of dispersion for most variables over time, as boxplots (Supplementary Fig. 6) and corresponding dispersion measures suggest (i.e., changes in median absolute deviations computed per variable and cluster, between the 1910 and 2010 cross-sections, Supplementary Table 3). By conducting Dunn’s test of pairwise comparisons for cross-sectional variable distributions within clusters, we also found increasingly fewer significant differences between medians per cluster, particularly for form variables within size-based clusters (Supplementary Fig. 7). From a multivariate perspective, within-cluster variance increases over time, in particular for form variables within size-based clusters Fig. 4d, e). This indicates that cities of similar size characteristics exhibit increasingly distinct urban forms. This decoupling of size and form-related attributes over time reflects that in 2010, there is more heterogeneity related to form in heavily developed than in less developed MSA clusters (Fig. 4b). This result suggests that the observed, systematic patterns regarding size do not apply as seamlessly to the form characteristics of MSAs, but rather follow more complex relationships.

### Regional distribution of thematic clusters.

Do these urban spatial forms and trajectories have an identifiable geography? If these thematic clusters also cluster in space, it could suggest that either the regions themselves or the timing of development may be driving differences in the spatial organization of MSAs. We examine this question by mapping the thematic clusters from 2010, 1960 and 1910 (Fig. 5d-f and j-l, respectively). The first clear pattern we observe is that the size-related clusters follow the well-known pattern of historical regional development of the United States. We find a regionally distinct grouping of already developed, larger MSAs in 1910 (Fig. 5f) along the eastern and western seaboard and the Northeast-Midwest corridor. The cluster represented by smaller points in 1910, in contrast, encompasses regions that were less developed in 1910 but experienced substantial urbanization over the following century. Through the mid-twentieth century, development spread from these traditional urban areas to the South and the Southwest (Fig. 5d, e).

While the size-related clusters closely follow well-known historical regional development trends in the US, the spatial patterning of the form clusters deviate from these trends, particularly from 1960. In 1910, the most notable pattern among the form clusters is the distinction between the areas spanning from the Northeast into the Midwest and the rest of the country (Fig. 5l). In general, the urban core of all MSAs in 1910 is relatively small (i.e., low MAXPATCHPROP). Where MSAs differ, however, is in their number of patches and their scatteredness. Reflecting very low levels of development and sparse settlements, areas outside of the Northeast-Midwest corridor score low on both of these measures.

We observe new types of urban development with a strong regional geography in comparing 1960 to 2010 (Fig. 5j, k). MSAs in the blue, square cluster (characterized by a high proportion of built-up area contained in a contiguous patch, i.e., high MAXPATCHPROP) emerging after 1985 (Fig. 4b) are concentrated in the Northeast-Midwest corridor, the Southeast and the Southwest. These areas with their dominant cores stand in contrast to MSAs in the less urbanized regions of the Plains, the South and Appalachia. Plains and Appalachian MSAs exhibit higher levels of scatteredness and patchiness, likely because of their abundant rural settlements and satellite towns. Thus, the contemporary dominant forms of differentiation by structural characteristics have a strong geographic imprint. These MSAs can be categorized as those that have large, highly integrated urban areas (blue, high MAXPATCHPROP values), typically along the coasts or in the traditional industrial belt of the US, and those that tend to be in the middle of the country and which are more internally fragmented (green-yellow colors, i.e., high dispersion measures NUMPATCH and SCAT). Given the regional geography of these clusters, it is difficult to rule out the possibility that the interior MSAs of the US may continue to converge on the profiles of their coastal and Midwestern counterparts over the decades to come.

### Regional and temporal deviations in urban spatial development.

Up to this point, we have demonstrated that urban spatial development is characterized by increasingly complex relationships between size and form-related properties, and this development process has unfolded in different places at different times. This finding implies that MSAs have grown across varied physical, economic and cultural landscapes and during periods of different technological development. Thus, the growing complexity of the US urban system across existing MSAs could stem from differences in the location and timing of development. This part of the analysis focuses on regional differences in the spatial complexity of MSAs by decomposing each variable into a regional time series of median values Fig. 6a, b) and formally testing for statistically significant differences in the attributes of MSAs across regions using Kruskal-Wallis tests (Fig. 6d) and subsequent Dunn’s tests for pairwise comparison (Fig. 6e, f, see Methods section).

We find that across larger regions (derived from US census divisions herein referred to as "regions", Fig. 6c), MSAs follow similar development trajectories. The regional time series of our size and form attributes follow very similar development paths (Fig. 6a, b) with few exceptions. This result indicates that MSAs are becoming larger (BUAREA), denser (BUDENS), and less circular (CIRC), and are increasingly dominated by a large contiguous urban tract of land (MAXPATCHPROP) across the conterminous US. While these time series suggest that MSAs are developing along similar trajectories across most regions, we also observe a few distinct regional patterns with respect to form-related characteristics, heavily driven by differences between interior and early-developed regions of the US. These differences become most evident when comparing the North Central MSAs, which appear to maintain higher levels of scatteredness (SCAT) (West North Central) and lower levels of clusteredness (CLUST) (East North Central) than their counterparts elsewhere. Urban areas in these MSAs tend to be more dispersed and less compact than in other regions. Conversely, MSAs in the Mountain region exhibit lowest levels of SCAT and NUMPATCH, and highest levels of CLUST over time. These pronounced regional trends result in distinct form characteristics for most MSAs in the interior of the US as compared to their counterparts along the coast. Notably, we observe a decreasing trend in NUMPATCH in most regions, starting earliest (i.e., around 1970) in the Northeast of the US (see also animation in Supplementary Movie 1).

We use Kruskall-Wallis (KW) tests to examine which attributes account for differences in urban form over time and across regions (Fig. 6d). While KW tests show statistically significant differences between regions for all variables of interest and years, the associated KW *H*-statistic provides a measure of difference among multiple regions (see Methods section). These tests confirm our finding of dramatic declines in differences in size attributes across regions (i.e., decreasing *H*-statistics) and persisting heterogeneity in the form attributes (i.e., relatively constant *H*-statistics, also reflected in the heterogeneity of median trends per variable and region, as shown in Supplementary Fig. 8). Consistent with the prior results, differences in the clusteredness and circularity of MSAs (measures of compactness) appear to account for much of the differences across regions.

While variation in size and density has sharply attenuated across regions and time, differences in form are more persistent. This result is evident in the regional variable-by-variable significance plots, which show very substantial declines in significant differences in size-related variables between regions from 1910 to 2010 (Fig. 6e, f). Our finding further underscores the strong regional unfolding of urbanization across the US. The increasing urbanization of the Southeast and Southwest in the second half of the twentieth century has produced a more even picture of urban size across US regions. In contrast, the form attributes depict a less pronounced decline in regional differences (Fig. 6f). However, much of these persisting differences in form are driven by the distinctive development patterns of the West North Central, West South Central and Mountain regions relative to the rest of the US.

### Ordinal relationships between size and form.

Several of our urban-spatial metrics are skewed (as indicated by low Shapiro-Wilk normality test scores, Supplementary Fig. 3), with some presumably approximating a power law distribution (e.g., BUAREA)^45-47^. Thus, we can gain additional insight by analyzing the relationships between size and form-related characteristics using ordinal scales (Fig. 7).

We calculated Spearman’s rank correlation coefficient between all size and form-related variables in 1910 and in 2010 (Fig. 7a and b), respectively. These results, along with their corresponding *Q-Q* plots (Fig. 7c and d), reveal further detail about the relationship between size and form attributes. While we observe high levels of association between dispersion measures (i.e., SCAT, NUMPATCH) with most size-related variables in 1910, many of the correlations attenuate by 2010, thus providing additional evidence for the disentanglement of size and form over time. However, we do also observe several notable exceptions to this finding. First, we observe an increasingly positive rank correlation between size characteristics and MAXPATCHPROP, which is driven by heavily developed MSAs that tend to exhibit greater connectedness and spatial contiguity. Secondly, we find stationary (negative) rank correlations between size variables and circularity, most notably between BUAREA and CIRC. This result indicates that large places tended to be less compact (i.e., less circular) than small places in the early 20th century, and that this trend is still evident in 2010. Even though this analysis does not explicitly test allometric relationships, these results are suggestive of such relationships between size and form-related urban-spatial metrics (see also animations in Supplementary Movies 2 and 3); formally testing these relationships over time is an important area for future research.

## Discussion

This study leverages information from HISDAC-US to examine urban spatial development in the United States since 1910. Irrespective of where MSAs are situated or when they started to grow, we find strong congruence in their size-related spatial development. That is, we find that more recently developing MSAs have followed similar paths to early-developing MSAs, particularly in terms of their size and density attributes. Even though urbanizing (i.e., not shrinking) MSAs follow more elaborate development trajectories in terms of structure, we also find evidence for strong grouping effects along these dimensions. This finding points to a set of increasingly complex urban forms, which have been emerging across the US. From a broad perspective, we find high degrees of continuity in the spatial development of urbanizing MSAs regarding size-related characteristics, which are quite robust to the timing and location of development.

Although urbanizing MSAs do follow similar size-related trajectories, there are some very notable exceptions to these overarching trends. Firstly, MSAs appear to be increasingly stratified, but less extremely distributed based on their size. This pattern is evident from cross-sectional normality tests (i.e., increasing Shapiro-Wilk normality test statistics for size variables over time, Supplementary Fig. 3) and from our cluster analysis, which indicates that while differences in city size have narrowed across MSAs over time, they are sorted into an expanding number of clusters or “types.” Furthermore, the form-related attributes of MSAs are growing increasingly independent from their size-related attributes. Secondly, although we find that, irrespective of location or timing of development, most urban spatial attributes are shifting in similar directions, there are some notable deviations across US regions. Most notably, MSAs in the interior census divisions of the United States follow urban forms that are quite different from coastal MSAs. Whether or not this is a temporary transition or indicative of newer and longer-lasting regional differentiation remains to be seen.

What do our findings tell us about the existence of systematic development trajectories across cities and their surroundings? Generally, our results suggest that urbanizing MSAs in the US appear to follow similar development trajectories irrespective of whether they developed recently or farther back in time with regards to size attributes. However, our multidimensional analyses reveal that these trajectories can be highly complex. While the source of this local heterogeneity is beyond the scope of this paper, the data and approaches put forward here provide a firm basis from which to examine these patterns, albeit subject to the issues associated with retrospective data.

**Fig. 1:**
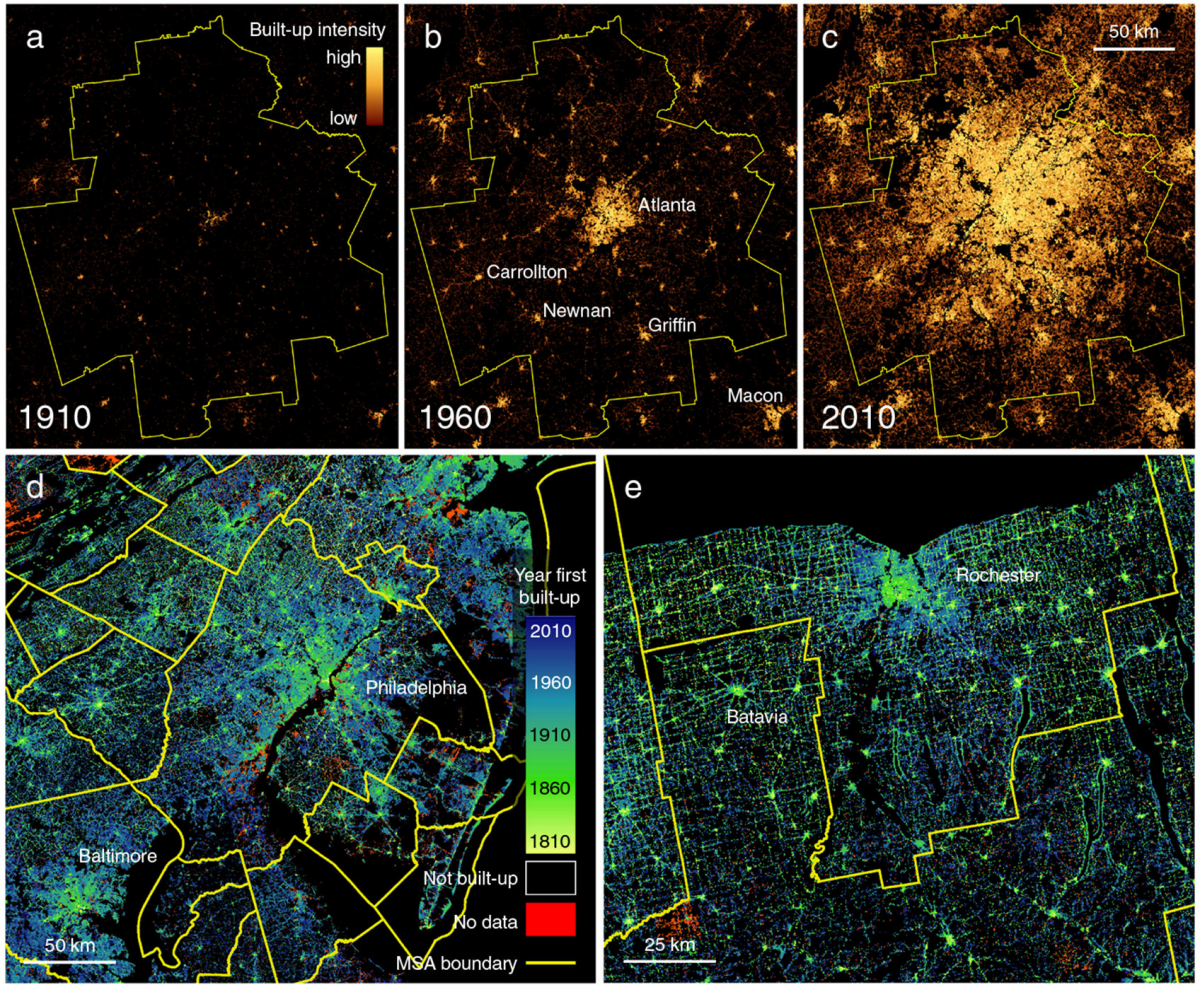
Gridded surfaces from the historical settlement data compilation for the US (HISDAC-US) used in this study. Built-up intensity depicted for the greater Atlanta metropolitan statistical area (MSA) in **a** 1910, **b** 1960 and **c** 2010. First built-up year composite shown for **d** greater Philadelphia and **e** for greater Rochester (New York). All datasets are at a spatial resolution of 250 × 250 m. Yellow lines represent MSA boundaries obtained from the US Census Bureau^51^, used as the primary analytical unit for this study.

The more detailed aspects of our findings also highlight the potential value of applying spatial scientific analysis to questions in urban history. Notably, major urban spatial transitions, such as post-WWII suburbanization^43^, and the increasing formation of interconnected, “megapolitan” city systems^48^ are evident throughout our results. Fig. 1, for example, shows that from approximately the late 1950s, average housing size and total number of properties sharply increased while measures of spatial compactness and circularity began to quickly decrease. These patterns reflect the sprawl of metropolitan areas beyond their central cities and into the emerging suburbs of the time. The emergence of “megapolitan areas” in the US is, for example, reflected in the emergence of the MSA cluster that is characterized by high levels of contiguity (see e.g., Fig. 5g). Historical developments such as these, which have been and will be the focus of ongoing research efforts^31,49^, undergird many of the broader patterns we observe here. While we do not attempt to grapple with these issues in this study, the approaches we articulate provide vast opportunities for enhanced understanding of the complex social, political and economic interactions that have brought about widespread historical spatial change.

City boundaries naturally change over time. In our analysis, we rely on fixed, retrospective boundaries of metropolitan statistical areas as they existed in 2010. While they suffer from a certain degree of arbitrariness (cf. Fig. 1), they allow for analysis within temporally consistent spatial units. However, the time series of certain structure- or density-based measures would be different when using temporally adaptive boundaries, and thus, future work could examine how our picture of urban spatial change would differ if we instead used a changing urban footprint or definition. Moreover, future work should include the analysis of smaller cities and towns defined as Micropolitan Statistical Areas (i.e., cities of less than 50,000 inhabitants) and expansion of this work to a more complete range of large and small cities in the US, and should also focus on the quantification of survivorship bias in the underlying ZTRAX data at the building level, e.g., by integrating auxiliary historical data sources.

Furthermore, forthcoming work should include additional spatial and temporal characteristics (e.g., differential measures such as expansion and densification of urban areas^31^), measures of intra- or peri-urban land use and vegetation, urban road networks, and census data (e.g., population, migration and other socio-economic variables). These data integration approaches in combination with advanced machine learning methods could enable predictive modelling of urban development and population changes. The integration of population data and remote sensing data will also allow for a quantification of the bias introduced into such analyses by the lack of information on building teardowns and urban shrinkage. The shrinkage phenomenon affects certain regions in the US and is typically associated with population decline and land conversion from built-up to less developed land. Such analysis will contribute to a broader understanding of drivers of and interactions between human and environmental processes and urban dynamics. Future analysis can generate valuable knowledge as a foundation for complex simulative models predicting and projecting future development of urban areas, population, and the interactions within socio-environmental systems. Moreover, the long-term spatial data used herein will potentially allow for testing urban scaling laws over the long-term, and assessing the impact of cross-sectional versus longitudinal analytical concepts on the outcomes of urban change and scaling analyses.

This work provides data and blueprints for broad, long-term investigation into spatial differentiation across urban environments. This study establishes an analytical foundation to measure well-known processes of urbanization, such as sprawl, infilling and densification, over long periods of time and at scales meaningful for analytical and interpretational purposes, both longstanding limitations in urban studies. Moreover, our analytical framework and the underlying data provide valuable insight for city and regional planners, allowing for the identification and forecasting of fine and coarse development trajectories. Knowledge of these trajectories may be particularly valuable in tackling issues related to environmental change, transportation and inequality^49^. We demonstrate how innovative data derivatives can be used to quantitatively assess different urban characteristics and their changes at fine spatial and temporal granularity. Our analysis demonstrates the value of applying data-driven analytical methods to the exploration of large spatial-temporal settlement data in urban geography, which can enhance our understanding of the historical settlement of cities in the US and elsewhere.

## Methods

### US metropolitan statistical areas.

The US Office of Management and Budget defines a Metropolitan Statistical Area (MSA) as a larger commuting area containing at least one urban cluster or urbanized area with a population of at least 50,000. It is comprised of the central county or counties containing the urban core, including adjacent counties characterized by a high degree of social and economic interaction with the central county or counties, which is measured through commuting^50^. Hence, MSA boundaries are spatial units containing urban cores, peri-urban areas and the urban fringe and thus constitute a suitable source of spatial zoning data for the analyses presented herein. In 2010, there were 363 MSAs in the conterminous US^51^ which were used in this study, and kept temporally consistent over the study period from 1910 to 2010.

### Gridded spatial layers.

Spatial layers consist of time series of gridded data layers at a spatial resolution of 250 m. These layers have been generated at semi-decadal temporal resolution for the study period 1910 to 2010, and include:

- **HOUS**: Count of housing units per grid cell and year^52^.
- **BUPROP**: Count of built-up properties (i.e., unique locations of housing units) per grid cell and year^53^.
- **Built-up intensity (BUI)**: Indoor area of all buildings per grid cell and year (unit: m^2^)^54^.
- **First built-up year (FBUY)**: Temporal composite containing the built year of the oldest structure per grid cell (unit: year)^55^.

These layers are available in the Historical Settlement Data Compilation for the US (HISDAC-US^28-30^, see Fig. 1 and Supplementary Fig. 9).

### Time series of descriptive variables per MSA.

The urban-spatial metrics used herein allow for a multi-faceted spatial perspective on urban systems, given the available data. We reduced an initial set of 50+ urban-spatial metrics to the 10 metrics used herein, based on a visual-analytical assessment involving plausibility, cross-correlation and variability analysis. Each of these variables is derived for each MSA and each 5-year interval for the study period 1910 - 2010, resulting in a multivariate time series characterizing the evolution of each MSA from different perspectives. We grouped these time series into two categories:

- **Size-related variables**: Characterizing the horizontal and vertical dimensions of urban systems, as well as the average size of the units comprising an urban system. In this category, we included density-based measures since they describe the relationship between horizontal and vertical extension of an urban system.
- **Form-related variables**: Characterizing the shape (i.e., geometric properties) of the urban footprint and its morphological structure (i.e., properties of the spatial entities constituting the urban footprint).

Similar categorizations are commonly used and suggested in urban studies^11,56^ and allow for separate analysis, as well as studying interactions between these categories. Below, we describe the urban-spatial metrics used, adjusted to the nature and the volume of our data.

#### Size related variables.

Based on zonal statistics of the spatial layer series within MSA boundaries (see processing workflow in Supplementary Fig. 9), we derived the following MSA-level time series characterizing the horizontal and vertical dimensions of urban systems^57^ and their interactions, guided by the information contained in our data:

- **BUAREA**: The planar area of the grid cells occupied by at least one building in a given year, as an absolute measure of horizontal extension of urban areas.
- **NETBUI**: The net built-up intensity, calculated as the total indoor building area in an MSA polygon in a given year. This measure allows for quantifying the total built-up volume^57^.
- **BUDENS**: The net built-up intensity per built-up ground area in an MSA in a given year. This measure relates to the commonly used floor-area-ratio (i.e., the quotient of building indoor area and area built upon, i.e., plot or parcel area^58^). However, due to the lack of footprint area or multi-temporal parcel area in our data, we used the area of the built-up grid cells as a proxy for plot area. By using the built-up area as denominator, rather than the MSA area, we overcome the issue of arbitrariness of MSA boundaries.
- **NUMBUPROP**: The number of built-up properties per MSA and year, an approximate, absolute measure of the size of the building stock in an MSA.
- **AVGHUSIZE**: The total BUI divided by the number of housing units within an MSA in a given year, as a measure of granularity of the units constituting the built environment.

#### Form-related variables.

Through segmentation and vectorization of the BUA layer series^59^, which was derived from the FBUY layer (see Supplementary Fig. 9), we obtained spatial vector objects of contiguous built-up areas within each MSA per year and derived the following variables:

- **NUMPATCH**: The number of built-up patches within an MSA per year, with a patch consisting of at least two grid cells, as a measure of fragmentation^60^.
- **SCAT**: The number of spatially isolated built-up grid cells (i.e., none of the 8 adjacent grid cells in a Moore neighborhood being built-up) within an MSA per year, as a measure of spatial scatteredness of the built-up area. This metric was adopted from the "scatter development"^61^ metric which represents the count of grid cells with less than 30% built-up area in their neighborhood. Since our BUI data measures total indoor area without specifying the area of building footprints, we chose to set this threshold to 0%.
- **MAXPATCHPROP**: The proportion of the largest built-up patch from the total built-up area within an MSA per year, as a percent-based measure of built-up area contiguity and dominance. This measure is related to the "largest patch index" (LPI)^60^. However, while LPI measures the proportion of the largest patch area with respect to the landscape area (i.e., the MSA area), we decided to use the total built-up area as denominator, in order to be independent from the (arbitrary) MSA area.
- **CIRC**: The circularity of the ten largest built-up patches per MSA and year. There are several approaches for measuring spatial compactness such as circularity in the geospatial sciences^62^; for example, the commonly used CIRCLE metric, relying on comparing the area of a shape to the area of its circumcircle^63^. Herein, we used the isoperimetric quotient, a commonly used circularity measure^64,65^ defined as the ratio of a polygon’s area *A* to the area of a circle with the same perimeter *p* as the polygon, in percent:
  (1) $$\text{CIRC}=100*\frac{4\pi A}{p^{2}}$$
  which we employed as an alternative measure of circularity, assumed to be computationally inexpensive to process the large amount of built-up patches in this study (>250,000 patches in all MSAs in 2010). Thus, CIRC characterizes the compactness of the largest patches constituting an urban system and is used as a measure of spatial complexity.
- **CLUST**: The clusteredness of built-up grid cells per MSA and year. This measure is the average nearest neighbor index (ANNI) based on the centroids of built-up grid cells. ANNI compares the observed average nearest neighbor distance ${\bar{d}}_{\text{NN,O}}$ of a set of point locations to the expected average nearest neighbor distance ${\bar{d}}_{\text{NN,E}}$ in a random point distribution of the same number of points and spatial extent^66^ and has been applied for long-term urban change studies and other spatial analyses^67,68^. The ANNI is calculated as:
  (2) $$\text{ANNI}=\frac{{\bar{d}}_{\text{NN,O}}}{{\bar{d}}_{\text{NN,E}}}$$
  with small values corresponding to highly clustered point patterns. Thus, we calculated our measure of clusteredness CLUST by subtracting the ANNI from the global maximum ANNI across all MSAs and points in time, to obtain large values for highly clustered point patterns:
  (3) CLUST=max(ANNI)−ANNI
  Supplementary Fig. 9 illustrates the performed geoprocessing steps to generate time series of these urban-spatial metrics.

### Data uncertainty handling

#### External validation of HISDAC-US source data.

The quality of built-up areas extracted from the HISDAC-US spatial layer series may suffer from missing housing records or from spatial offsets in the geolocations contained in the ZTRAX database, causing grid cells falsely labelled as "not built-up". To quantify these positional uncertainties, we conducted a multi-temporal accuracy assessment against a highly accurate reference database created from cadastral parcel records and building footprint data in 31 US counties, yielding *F*-measures of 0.9 or higher for each evaluated year in the time period 1910 - 2010^29^. Moreover, we conducted a US-wide accuracy assessment of built-up areas in 2016 derived from the HISDAC-US data against a remote-sensing derived building footprint dataset, yielding similarly high accuracy values in urban areas^30^. Furthermore, we cross-compared temporal trajectories of housing unit counts (HoUS) against census-based housing statistics^30^.

#### Incompleteness of temporal information and locational uncertainty.

As shown in^29^ the HISDAC-US inherits issues of data incompleteness from the original ZTRAX data. For example, 82 counties (i.e., approximately 2.5% of the land area) in the conterminous US do not contain any settlement-related ZTRAX data records. Approximately 20% of the built-up areas in the conterminous US are lacking temporal information (i.e., the year when a structure has been built) necessary to map built-up areas at a given point in time. However, the large majority of these areas is located in rural regions^30^. In this study, we excluded MSAs that have an area proportion of more than 5% of a county of known data missingness. Furthermore, we excluded MSAs that have a temporal missingness of more than 50%. Besides data missingness, there are issues due to generalization of the geospatial locations when dummy coordinates are used (i.e., the use of identical coordinates for numerous settlement locations within an MSA). In order to account for this, we computed the ratio between the NUMBUPROP and HOUS layers in 2010. We excluded the MSAs exceeding the 95th percentile of this ratio, i.e., MSAs containing extremely high proportions of housing units assigned to an identical geolocation, indicating the presence of dummy coordinates. In total, 31 of 363 MSAs were excluded from this study (see map of MSA-level data completeness, Supplementary Fig. 10).

#### Time series correction.

For the remaining MSAs, we developed a correction procedure applied to the time series that works as follows: We created a spatial layer indicating the indoor area, the number, and the presence of properties per grid cell without built year information^29^ and added those grid cell values to the contemporary layers, BUI_2015_, NUMBUPROP_2015_, and BUA_2015_, respectively. The key assumption is that buildings without built year information exist in 2015. Based on these corrected layers, correction terms are calculated and the variables are recomputed for each MSA in 2015. We use the resulting correction terms, proportionally, to adjust the variable values in the previous years, while preserving the relative change between two subsequent points in time (see^31^ for details). We evaluated the accuracy of this correction procedure in an experiment in which we artificially removed a random sample of up to 50% of the temporal information from MSAs of originally high built year attribute completeness, and compared the original and the corrected time series per variable and across time. Boxplots in Supplementary Fig. 11 shows the relative error distributions over time, based on *N* = 100 realizations of the described procedure. We found high accuracies in our corrected time series for the MSAs under test for the time period 1910–2010.

#### Temporal mismatch and survivorship bias.

**Fig. 2:**
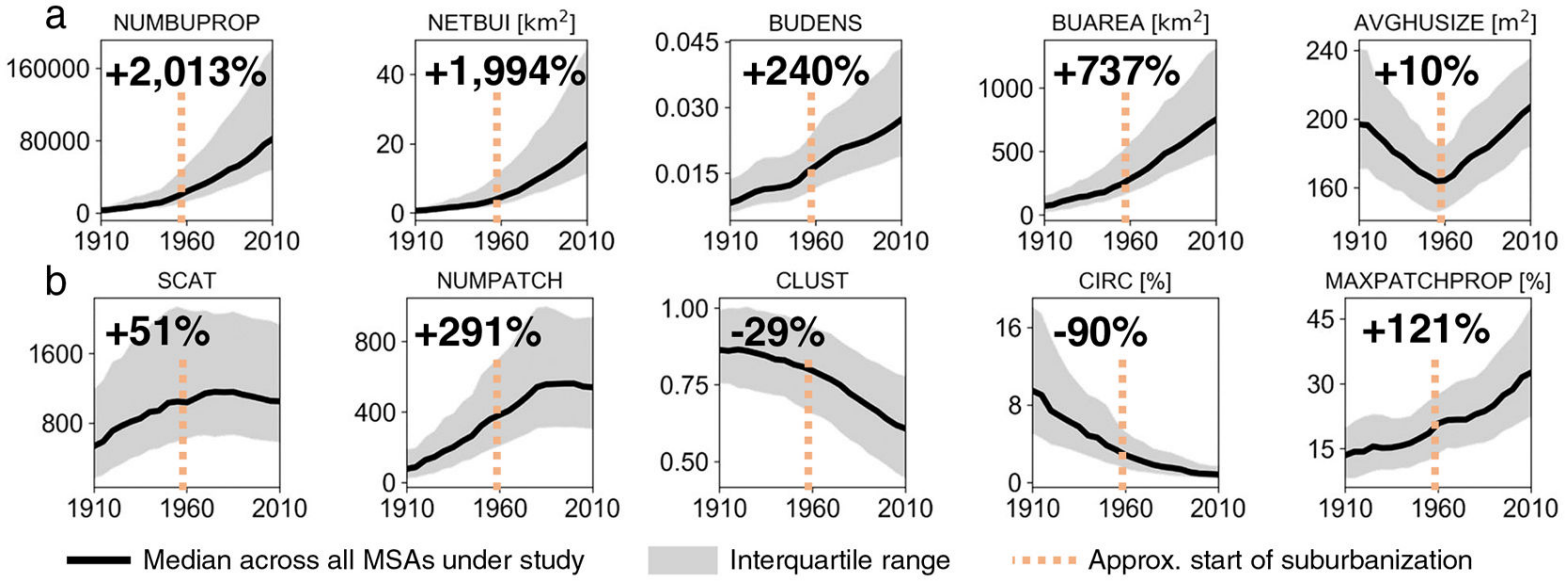
Temporal trends of the analyzed urban-spatial metrics across all metropolitan statistical areas. Median (black) and interquartile range (grey) of the variables characterizing **a** size and density, and **b** shape and structure, over all analyzed metropolitan areas. Percentages depicted represent the change in median of the respective variable from 1910 to 2010. Also shown is the approximate start of US suburbanization in the late 1950s (vertical dashed lines in red).

Neither does the ZTRAX database contain information on teardowns or building replacements, nor does it provide sufficient information on remodeling activities. Thus, attributes of built-up structures may refer to a contemporarily existing building and may not reflect the characteristics in the year when a location became first built-up (i.e., the built year information on record). This may result in a selection bias, or survivorship bias, in a sense that information on first settlements, typically small buildings, has been replaced by the characteristics of larger buildings that replaced the original buildings. This issue is also seen as a selection bias, introduced by the nonrandomness of building replacements, e.g., the survival of a building depends heavily on its characteristics (size, material), and the time period in which it was built^40,41^. This effect likely causes a biased view on the building stock in early years, and cannot be easily quantified nor mitigated, thus remaining the largest source of uncertainty in this study. The lack of information on teardowns and building replacements likely causes an increasing underestimation of the building stock towards early points in time which can, to some degree, be measured by comparing our results to population data. We carried out such a comparative analysis and found that the overall trends reported herein are largely unaffected by this latter issue. To do so, we obtained decadal census population counts per MSA since 1940, and calculated the average population-building ratio (PBR) per MSA and year. We compared these ratios to census-based average population-household ratios (PHR) and observed a similar decreasing trend for both PBR and PHR over time (Supplementary Fig. 2a), although PBR has much larger values than PHR in earlier years. While these dynamics may be a superposed effect of a variety of processes, and the differences may partially be the result of different reference units (i.e., buildings versus households) and geographic coverages (MSAs versus all US including rural areas), we partially attribute the divergence observed in earlier years to underreporting in the ZTRAX data. We used the maximum PBR (PBR_max_) per MSA, calculated over all years, as an MSA-level measure of underreporting. While the PBR_max_ is not a function of absolute MSA size, measured by NETBUI_2010_, as Supplementary Fig. 2b suggests, we apply a range of thresholds to PBR_max_ and assessed the sensitivity of the overall trends reported in Fig. 2. Results suggest that these overall trends are mostly unaffected by early-year underreporting in ZTRAX. That is, even MSAs with PBR_max_ below the 5th percentile, and thus, least affected by underreporting, exhibit similar trends compared to the baseline trend using all MSAs. (Supplementary Fig. 2c).

#### Sensitivity to spatial resolution.

The urban spatial metrics used herein may be sensitive to the spatial resolution of the underlying gridded data layers. To assess whether the observed trends hold across different spatial resolutions, we randomly selected 25 of the 300+ MSAs and computed the metrics for a range of cell sizes (i.e., 30 m, 100 m, 400 m, 500 m) besides the cell size of 250 m used herein, and visualized the resulting trends for each cell size (Supplementary Fig. 1). Despite different magnitudes for some metrics, we observe relatively similar trends over time, and find that the chosen resolution of 250 m represents an acceptable tradeoff between spatial generalization (i.e., capturing most components of the urban environment, such as impervious surfaces) and uncertainty, while preserving the characteristic shapes of urban extents.

### Analytical methods

#### Cross-temporal quantitative trajectory analysis.

**Fig. 3:**
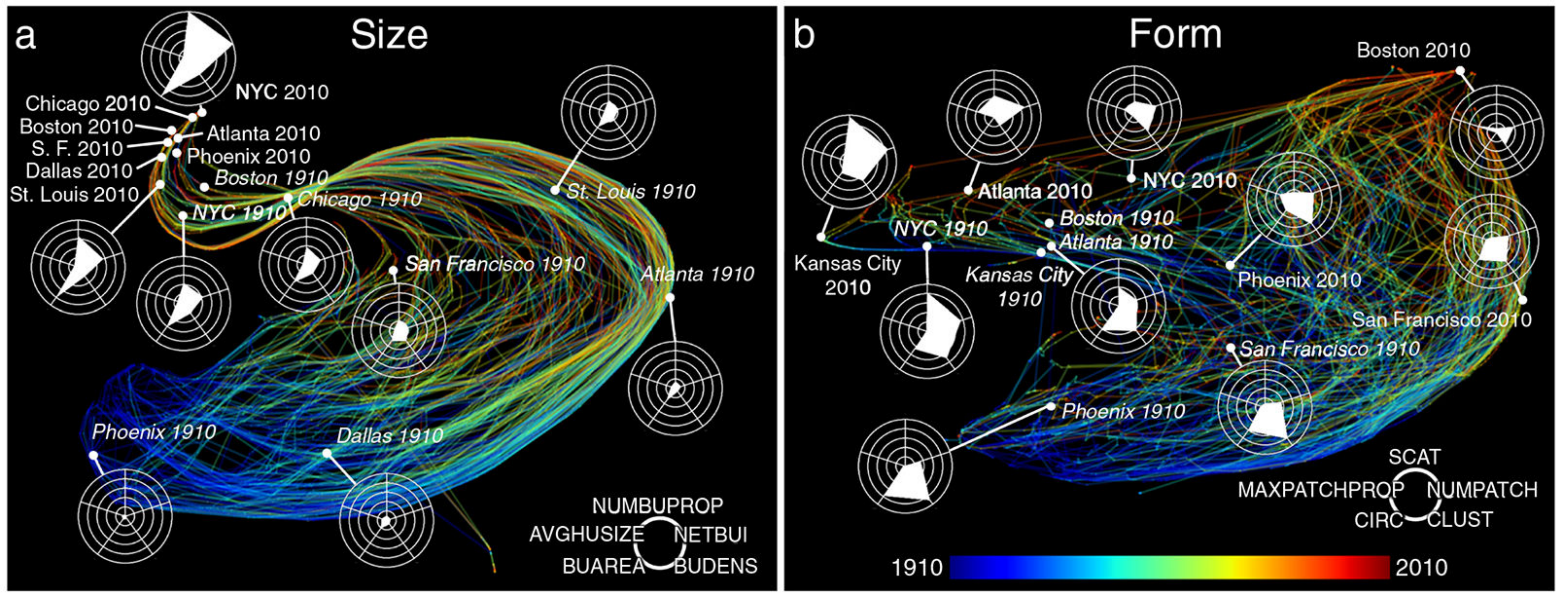
Cross-temporal trajectories of metropolitan statistical areas visualized using t-SNE. Trajectories are shown with respect to **a** size-related variables, and **b** form-related variables. Similar MSAs are depicted close to each other; similarity is visualized in two-dimensional attribute spaces using t-SNE dimensionality reduction. The vector of a trajectory represents the changes occurring in each MSA over our study period from 1910 (blue) to 2010 (red). Radar charts illustrate the underlying variables for selected MSAs in 1910 (italic) and 2010.

Each MSA at any given year can be represented as a point in a 5-dimensional attribute space defined by the variables derived for each of the two categories. In order to analyze similarity between the MSAs within each category, we used t-distributed stochastic neighbor embedding (t-SNE)^69^ to reduce the dimensionality of these attribute spaces. T-SNE allows for mapping higher-dimensional data into low-dimensional spaces where Euclidean distance among nearby data points in the target space represents their similarity in the original space. We performed t-SNE for each category using the described variables extracted for each MSA and each year to visually assess the similarity between MSAs at different points in time in a two-dimensional space. See Fig. 3 and Supplementary Figs. 4, 5.

#### Variable selection and dimensionality reduction.

In order to reduce the complexity of the subsequent analyses and enable legible and comprehensive interpretation of the results, we decreased the number of variables to three per category using principal component analysis applied to the full data (i.e., all MSAs and all years). We retained those three variables per category that showed the highest factor loadings of the first principal component (PC1) (Supplementary Table 1). These variables represent the highest contribution to the variability in the respective data distributions.

#### Multi-temporal thematic cluster analysis.

**Fig. 4:**
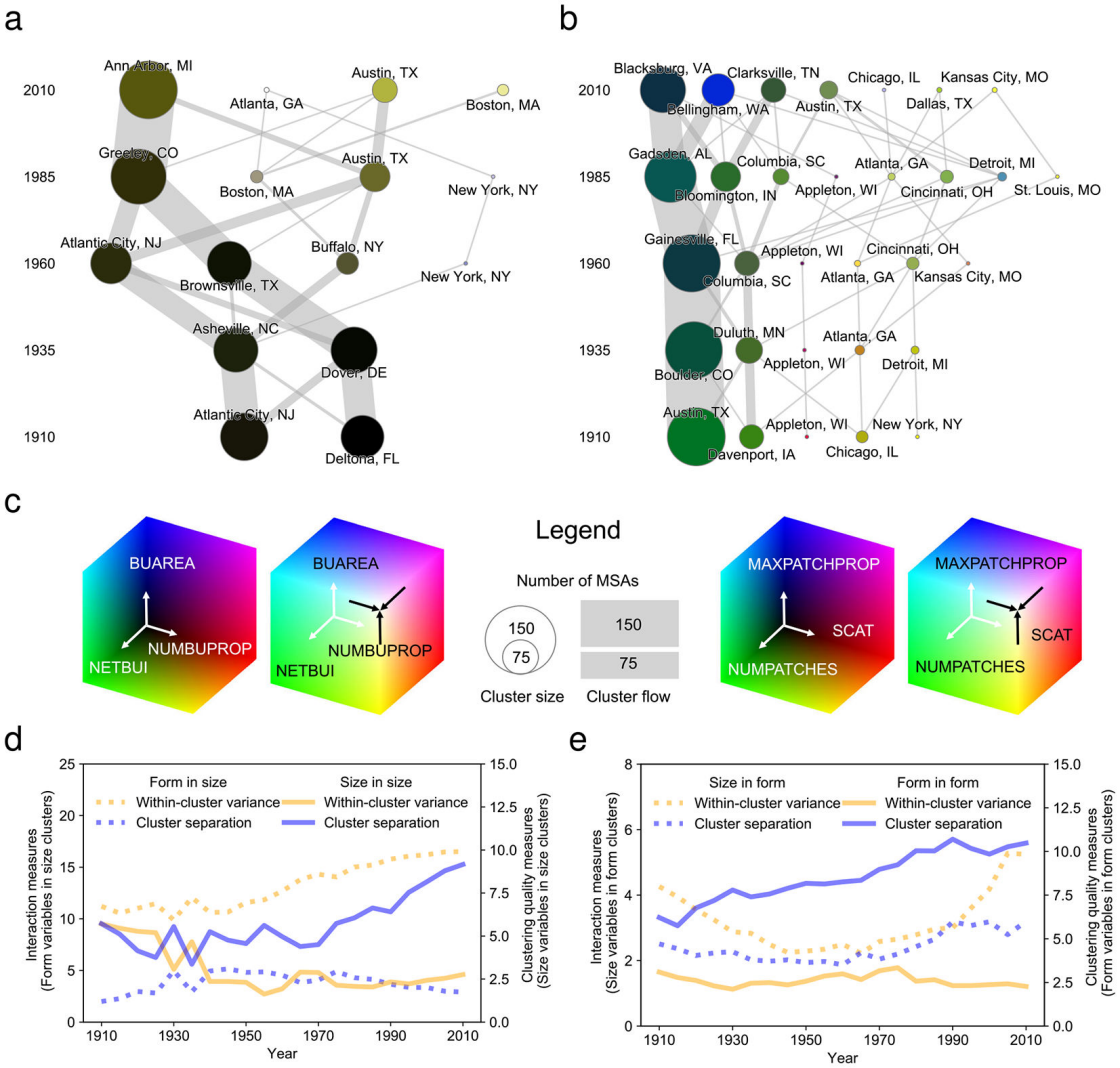
Cross-temporal cluster analysis, carried out for cross-sections of the MSA variables in intervals of 25 years. **a** Size-related variables (NUMBUPROP, NETBUI, BUAREA), and **b** form-related variables (NUMPATCH, SCAT, MAXPATCHPROP). Each node of the depicted networks represents a cluster of MSAs, node sizes correspond to the number of MSAs per cluster, and node colors represent the location of the cluster center (i.e., medoid) in the respective attribute spaces, using RGB color-coding. The legend for the attribute color-coding is shown in **c**. The width of the links (grey lines) between clusters identified in subsequent years represent the number of MSAs transitioning between clusters over time ("cluster flows"). Line plots show measures of cluster separation (blue) and within-cluster variance (orange) over time for: **d** size-based clusters, and **e** form-based clusters. Dashed lines show cluster separation and within-cluster variances for form variables in size-based clusters (**d**), and vice-versa in **e**, indicating the disentanglement of size and form over time.

**Fig. 5:**
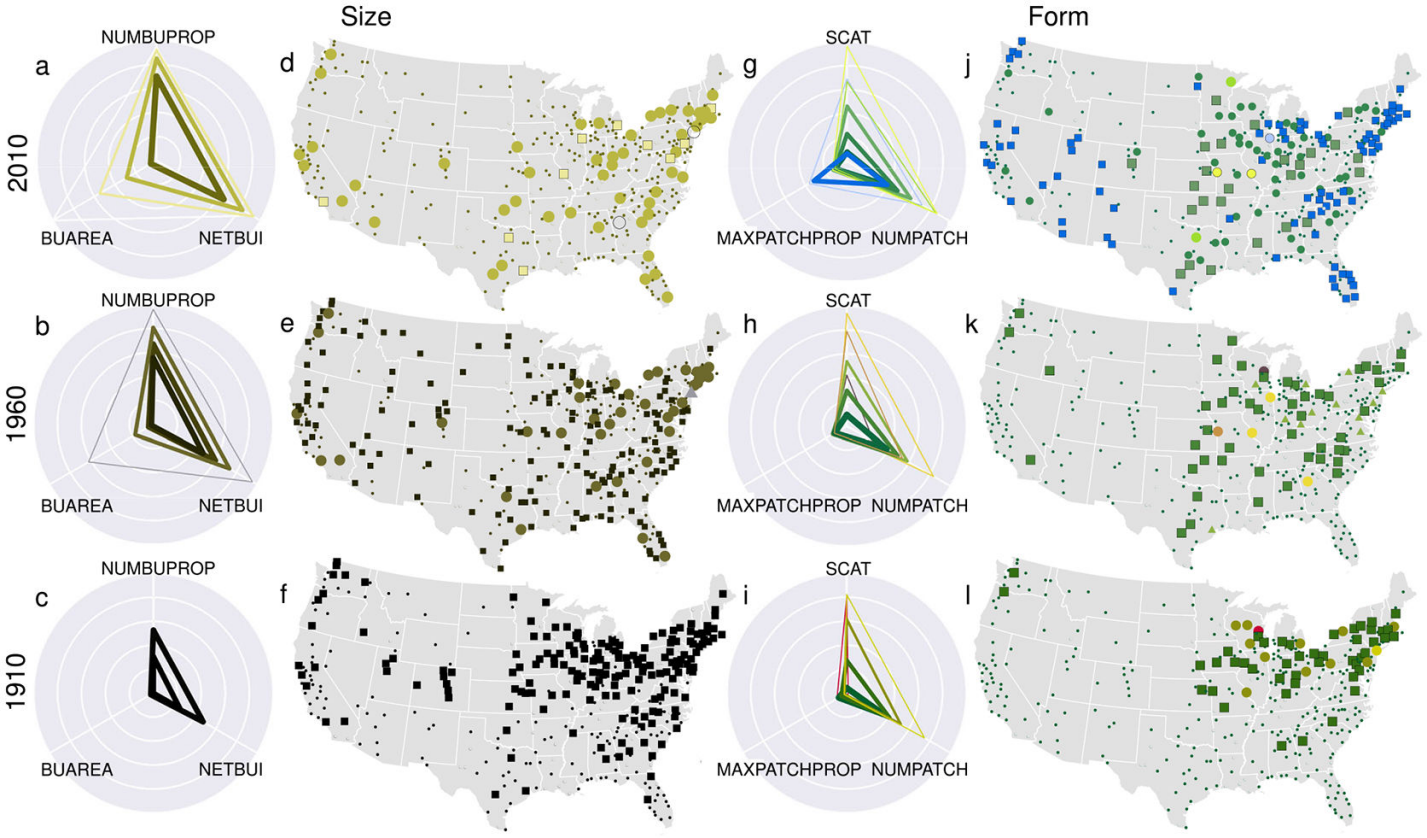
Characteristics and geographic distribution of the identified clusters of metropolitan statistical areas over time. Radar charts depicting the log-transformed characteristics of cluster medoid MSAs for the size characteristics in **a** 2010, **b** 1960, and **c** 1910. Line thickness represents the log-transformed cluster sizes (i.e., number of MSAs in each cluster); Panels **d–f** show corresponding multi-temporal maps of MSA cluster memberships, with clusters definied by color and shape. Colors in **a–f** correspond to the RGB color-coding used in Fig. 4a, b, see Fig. 4c for a corresponding legend. Panels **g–l** show the respective radar charts and maps for the form variables. Depicted locations are centroids derived from MSA boundaries obtained from US Census Bureau^51^, also shown are state boundaries (white) obtained from the US Census Bureau^75^.

Whereas the described t-SNE based trajectory visualization method reveals spatio-temporal MSA-level patterns of similarity and variability, they do not inform about the number, homogeneity, characteristics and size of thematic clusters and their variation over time. Thus, we developed a framework for cluster analysis of the temporal cross-sections of MSAs. For each point in time and each of the two variable categories, we identified clusters of MSAs using the BIRCH clustering algorithm (balanced iterative reducing and clustering using hierarchies), a tree-based clustering algorithm suitable for non-uniformly distributed data^70^. BIRCH allowed us to determine the number of clusters based on the data (rather than specifying a fixed number of clusters) and the size of each cluster for each temporal cross-section. We ran BIRCH without specifying a number of clusters, thus using the subclusters returned by BIRCH before the final, global clustering step when the user-specified threshold value is reached (i.e., the so-called tree-BIRCH method^71^). We adjusted this threshold empirically for each category of variables. Since the same threshold was used for all temporal cross-sections of the data, the resulting clustering sequences are independent from the threshold and intercomparable. The thresholds used as stopping criteria were 0.15 (size-related variables), and 0.125 (form-related variables). A branching factor of 50 was used for all cluster analyses. The resulting temporal sequence of clusters allowed us to assess how MSAs maintain or switch memberships between clusters in subsequent years. Finally, we registered the MSA representing the medoid of each cluster in the three-dimensional space spanned by the used variables in a given year, in order to derive MSAs typical for a cluster at each point in time (see Fig. 5). The variable magnitudes of these medoid MSAs were RGB color-coded for the visualizations of the clusters (see Fig. 4). Moreover, we visualized these clusters in geographic space for selected years in order to reveal spatial patterns of thematic clusters (see Fig. 5). We assessed clustering validity by generating time series of within-cluster variability and between-cluster variation^72^, and, likewise, used these metrics to evaluate distributions of size-variables within form-based clusters, and vice-versa (Fig. 4d,e). To further assess these interactions, we conducted Dunn’s test of pairwise comparisons on size variable distributions within form-based clusters, and vice-versa, for each point in time (Supplementary Fig. 7) and assessed within-cluster dispersion (Supplementary Fig. 6, Supplementary Table 3).

#### Regionalized time series generation.

**Fig. 6:**
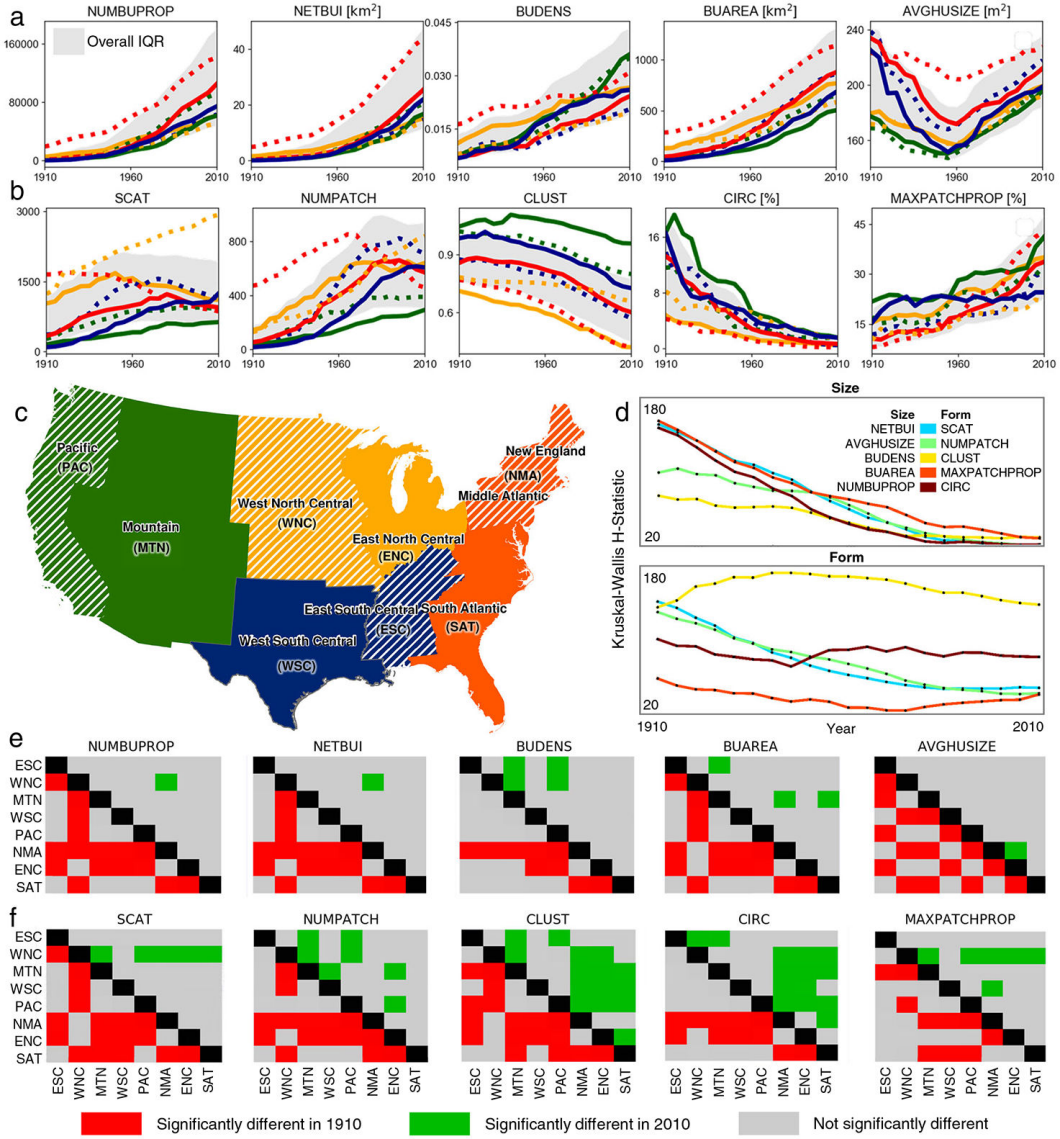
Regionally decomposed trends of urban spatial development. Median time series differentiated by region, **a** for size- related variables, and **b** for form-related variables; **c** map of regions derived from US census divisions, obtained from US Census Bureau^73,75^ (with New England and Middle Atlantic divisions merged), colors of regions corresponding to line colors in **a** and **b**, and hatched regions correspond to dashed lines in **a** and **b**; Panel **d** shows time series of Kruskal-Wallis H-statistics for each variable, and panels **e**, **f** show corresponding significance plots indicating statistically significant differences between specific divisions using Dunn′s Test of Multiple Comparisons for 1910 (red) and 2010 (green) for size variables and for form variables, respectively.

**Fig. 7:**
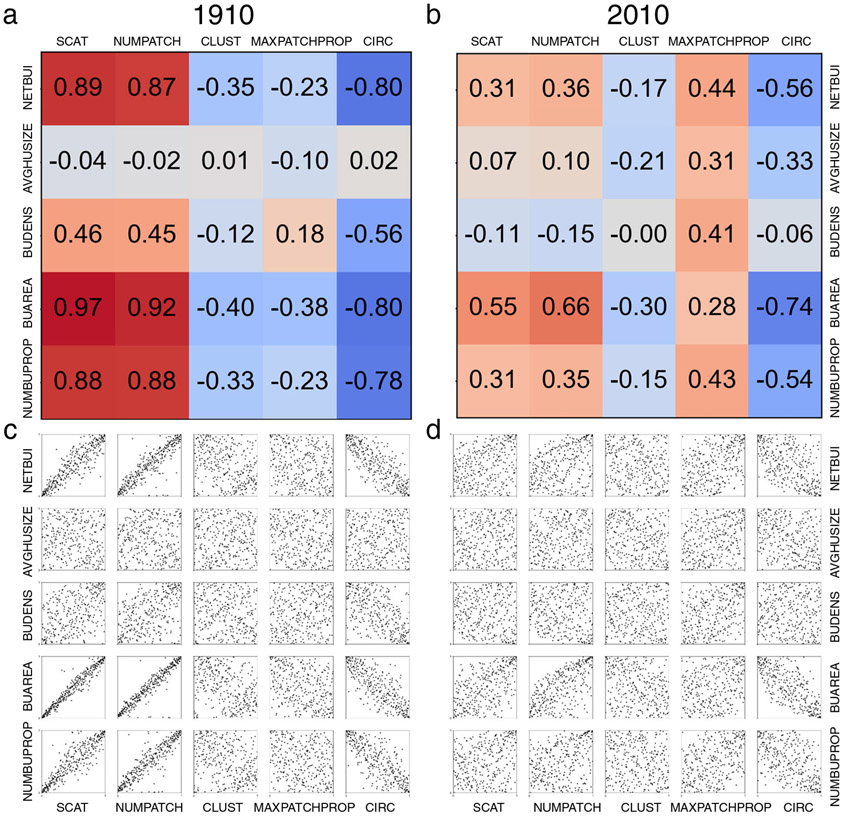
Assessing ordinal relationships between size and form-related characteristics over time. Heat maps of Spearman′s rank correlation coefficient between size and form variables **a** in 1910, and **b** in 2010, ranging from −1 (blue) to +1 (red). The bottom row shows corresponding Q-Q plots of corresponding pairs of MSA-level attributes **c** in 1910, and **d** in 2010.

We generated median time series per variable and geographic region in the US, adapted from the US census divisions^73^. Moreover, we used Kruskal-Wallis (KW) tests to analyze whether regional median time series exhibit statistically significant differences, and how statistical significance behaves over time. We used non-parametric KW tests since non-normality of most variables can be assumed based on Shapiro-Wilk tests applied to temporal cross-sections of the data (Supplementary Fig. 3). While it is common practice to use the p-value returned by the KW test as a measure of statistical significance, we also analyzed time series of the *H*-statistic returned by each KW test (Fig. 6d) as a measure of difference between group (i.e., region) distributions. Since the sample sizes per variable and year are identical (i.e., the number of MSAs included in the study), the *H*-statistic is based on the same degrees of freedom and thus, comparable across variables and over time. Moreover, we conducted subsequent posthoc Dunn’s test of pairwise comparisons per year to analyze between which pairs of regions the identified statistically significant differences occurred (see Fig. 6e,f).

### Limitations of this work.

Whereas the results are promising, several limitations of the presented work need to be mentioned: First, the ZTRAX database and the derived HISDAC-US data do not take into account teardowns and building renovations. Thus, built year information may refer to the first existing building, whereas other attributes, such as building size, may refer to the structure existent at present, allowing only for analyzing the "surviving" building stock. Moreover, this limits our analysis to the measurement and quantification of urban growth, and does not take into account the shrinkage of built-up areas, with Detroit as a popular example. This limitation may slightly distort variables that take into account age and built-up intensity in an integrated manner. However, as Supplementary Fig. 2 suggests, our main findings are largely invariant to these effects. Moreover, buildings in public lands may suffer from lower levels of coverage in the ZTRAX database. Second, some MSAs were excluded in this study due to data incompleteness (Supplementary Fig. 10). In the future, more sophisticated data correction methods (e.g., based on ancillary data and machine-learning based predictive models) will be employed to fill these data gaps more reliably, allowing for a complete analysis of urban evolution in the US using the proposed methods.

## Supplementary Material

Supplementary Information

## Figures and Tables

**Table 1:** Urban spatial metrics used in this study: The variables, calculated per MSA and year, used to describe the evolution of urban areas in this study, categorized into two categories (i.e., size and form). Variables in each category are sorted by their factor loadings from principal component analysis, conducted across all MSAs and points in time, indicating how much data variance these variables explain (sorted per category, descending from top to bottom), see Supplementary Table 1.

Variable	Characteristic of built-up areas	Description
**Size**		
Built-up area (BUAREA)	Horizontal extension	Total built-up area
Number of built-up properties (NUMBUPROP)	Quantity of built-up elements	Number of built-up properties
Net built-up intensity (NETBUI)	Built-up volume	Total indoor area of buildings within an MSA
Built-up density (BUDENS)	Built-up density	Total indoor area of buildings per built-up area)
Average housing unit size (AVGHUSIZE)	Built-up element size	Average size of housing units, measured in indoor area
**Form**		
Scatteredness (SCAT)	Dispersion	Scatteredness: number of isolated built-up grid cells
Number of built-up patches (NUMPATCH)	Dispersion	Number of contiguous areas (patches) of built-up land
Largest patch area proportion (MAXPATCHPROP)	Contiguity / dominance	Largest built-up patch area proportion of total built up area
Clusteredness (CLUST)	Compactness / dispersion	Deviation of built-up cells from a random spatial distribution
Mean circularity (CIRC)	Compactness	Circularity of the ten largest built-up patches

## Data Availability

The HISDAC-US geospatial data layers used for this study have been made publicly available under https://dataverse.harvard.edu/dataverse/hisdacus. Moreover, the urban spatial metrics per MSA and year are available at https://doi.org/10.6084/m9.figshare.13303091^74^.
