# Supplementary material for: A century of decoupling size and structure of urban spaces in the United States
Source: Commun Earth Environ. Author manuscript; Available in PMC 2021 Dec 29. (PMC8716013; doi:10.1038/s43247-020-00082-7)
Supplement: Supplementary Information [file NIHMS1761691-supplement-Supplementary_Information.pdf]

Supplementary Information for: A century of decoupling size and structure of urban spaces in the United States

Johannes H. Uhl, Dylan S. Connor, Stefan Leyk, Anna E. Braswell

Supplementary Materials - Overview

Supplementary Table 1. Variable glossary and PCA results

Supplementary Fig. 1. Sensitivity analysis of time series to spatial resolution

Supplementary Fig. 2. Sensitivity analysis of time series to early-year underreporting

Supplementary Fig. 3. Multi-temporal Shapiro-Wilk normality tests

Supplementary Fig. 4. Regionally decomposed t-SNE-based MSA trajectories (size variables)

Supplementary Fig. 5. Regionally decomposed t-SNE-based MSA trajectories (form variables)

Supplementary Table 2. Complexity quantification of MSA trajectories in t-SNE space

Supplementary Fig. 6. Variable distributions within identified clusters

Supplementary Table 3. Quantification of variable dispersion within identified clusters

Supplementary Fig. 7. Significance plots of medians of distributions within identified clusters

Supplementary Fig. 8. Regionally decomposed temporal trends for each variable

Supplementary Movie 1. MSA bar chart race animation for the NUMPATCH variable (1910 - 2010)
`https://public.flourish.studio/visualisation/2765858`

Supplementary Movie 2. MSA bar chart race animation for the NETBUI variable (1910 - 2010)
`https://public.flourish.studio/visualisation/2765807`

Supplementary Movie 3. MSA bar chart race animation for the BUAREA variable (1910 - 2010)
`https://public.flourish.studio/visualisation/2765392`

Supplementary Fig. 9. Data processing workflow diagram

Supplementary Fig. 10. MSA-level completeness of temporal information

Supplementary Fig. 11. Time series correction validation results

Supplementary References

Supplementary Table 1: Variable glossary and PCA results. The source data reference indicates the underlying dataset from the HISDAC-US data repository. Also reported are eigenvalues, factor loading and Pearson's correlation coefficient for first principal component of a PCA across all MSAs, variables, and years, separately for size and form related variables. Top three variables (based on PC1 factor loadings) were used for cluster analysis.

Variable w/ source data reference	Short name	Measured characteristic	Unit	Technical description	Eigenvalue	PC1 factor loading	Correlation to PC1
**Size**							
Built-up area [1,2]	BUAREA	Horizontal extension	km^2	Area of grid cells with at least one built-up structure	0.0294	0.0835	0.9639
Number of built-up properties [3]	NUMBUPROP	Quantity of built-up elements	-	Number of built-up properties	0.0833	0.0662	0.9708
Net built-up intensity [4]	NETBUI	Built-up volume	km^2	Total indoor area of all built-up properties within an MSA	2.9375	0.0526	0.9405
Built-up density [1,4]	BUDENS	Built-up density	-	Total indoor area per built-up area (BUAREA)	0.1657	0.0145	0.2833
Average housing unit size [4,5]	AVGHUSIZE	Built-up element size	m^2	Total indoor area by number of housing units	1.7841	0.0046	0.0976
**Form**							
Scatteredness [2]	SCAT	Dispersion	-	Number of spatially disjoint grid cells containing at least one built-up structure	2.5259	0.0942	0.6270
Number of built-up patches [2]	NUMPATCH	Dispersion	-	Number of spatially disjoint patches of built-up area	1.4039	0.0591	0.7124
Largest patch area proportion [2]	MAXPATCHPROP	Contiguity / dominance	%	Area of the largest built-up patch in proportion to the overall built-up area	0.1517	0.0256	0.3230
Clusteredness [2]	CLUST	Compactness / dispersion	-	Average nearest neighbor index of the centroids of all built-up grid cells	0.6848	-0.1316	-0.7829
Mean circularity [2]	CIRC	Compactness	%	Average circularity measure of the ten largest patches	0.2336	-0.1455	-0.8948

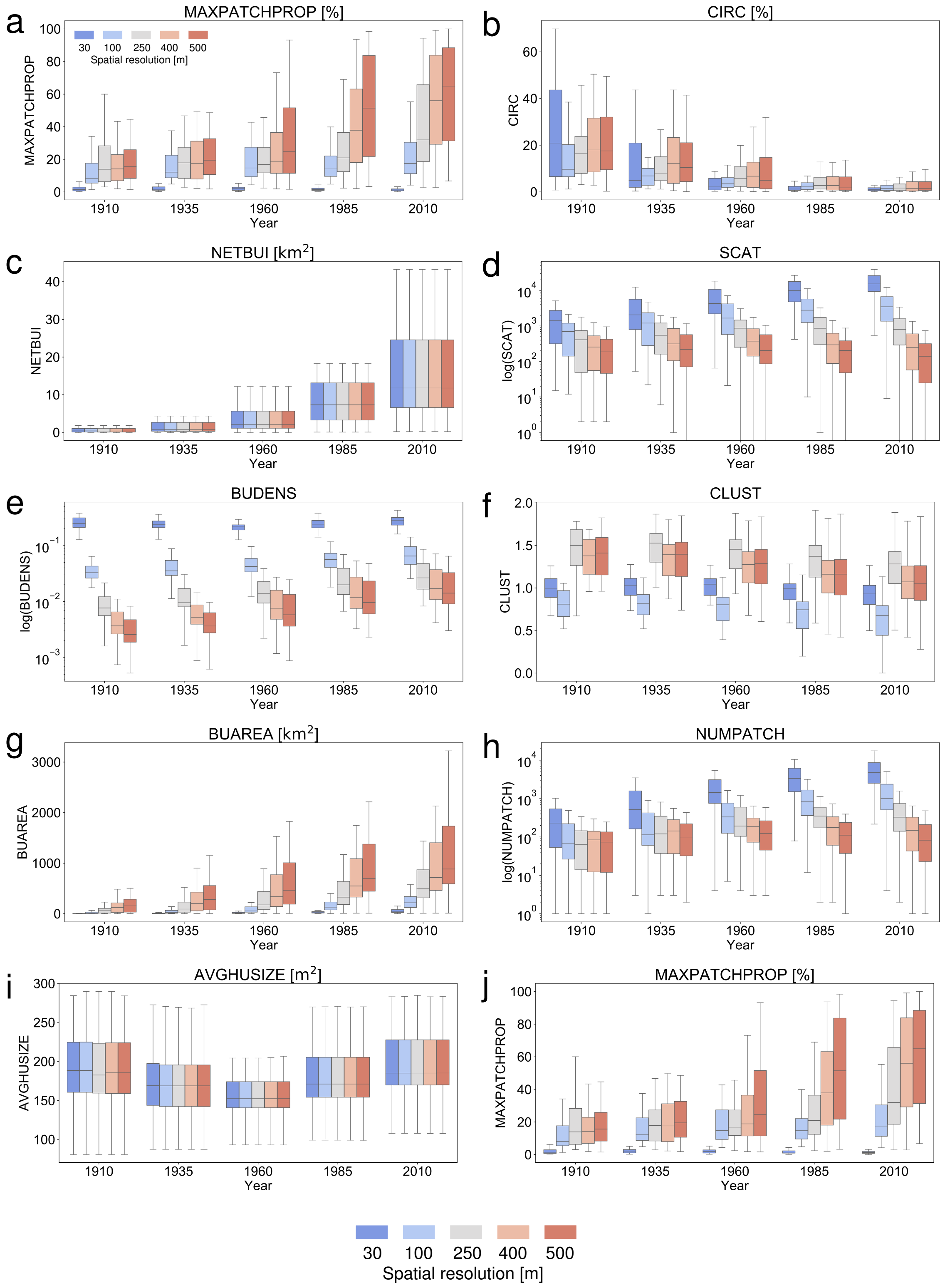

Supplementary Figure 1: Assessing the sensitivity of temporal trends to spatial resolution of the underlying gridded data for each of the 10 variables used. The gridded surfaces underlying each urban-spatial metric were resampled to a range of spatial resolutions, and temporal trends for each variable were extracted for each resolution. Distributions are based on a random selection of 25 MSAs. Some variables are log-transformed for visualization purposes. Box-and-whisker plots show medians (horizontal lines), lower and upper quartiles (boxes), and whiskers extend to $1.5 \times IQR$ (interquartile range) below the lower quartile, and above the upper quartile, respectively.

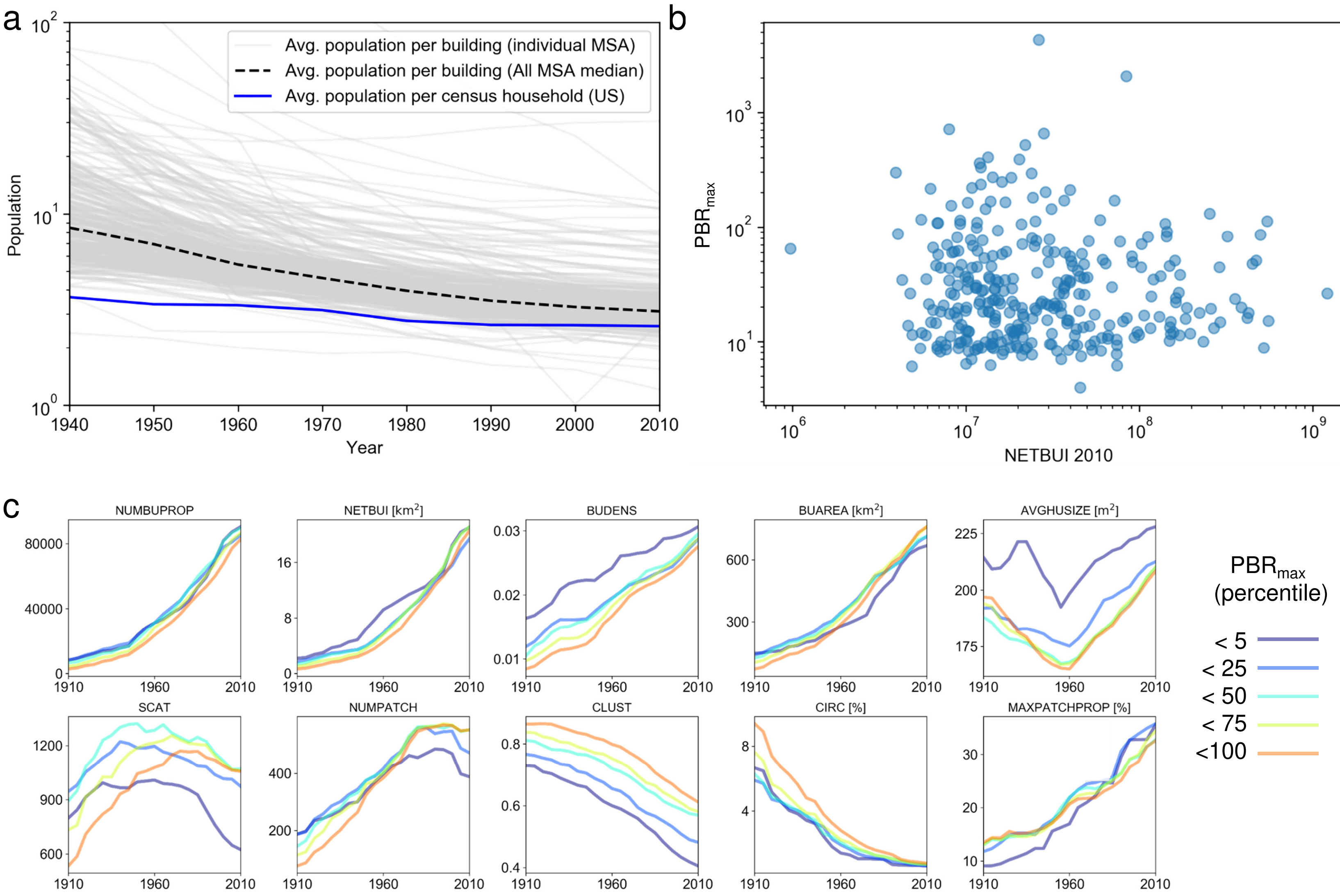

Supplementary Figure 2: Assessing the sensitivity of temporal trends to MSAs affected by building teardown or replacement activity, causing underreporting in the building stock towards earlier points in time: (a) Trajectories of population-building ratio (PBR) for each MSA, across all MSAs and average census-based population-household ratio across the US, (b) scatterplot of the maximum PBR per MSA across all points in time, against 2010 NETBUI, indicating no strong relationship between PBR and city size, and (c) overall (median) trends, as reported in main document Fig. 2, for various thresholds of PBR_{max}, illustrating the effects of building stock underreporting in early years on the observed general trends over time.

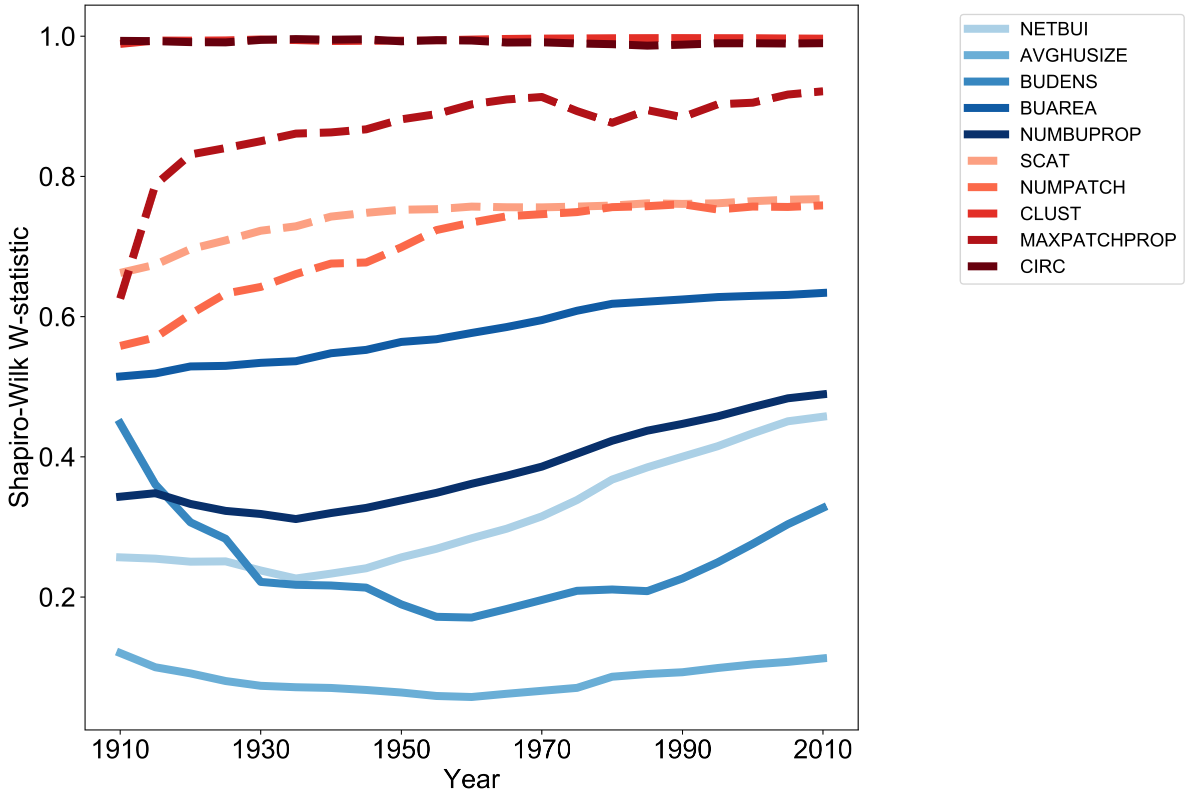

Supplementary Figure 3: Multi-temporal normality test of variable distributions: Time series of Shapiro-Wilk[6] normality test scores per variable. High values indicate higher degrees of normality. Dashed lines represent the shape-structure variables. Size-related variables appear to be more skewed than form-related variables, but exhibit less skewness over time, indicating less extreme outliers in city size distributions.

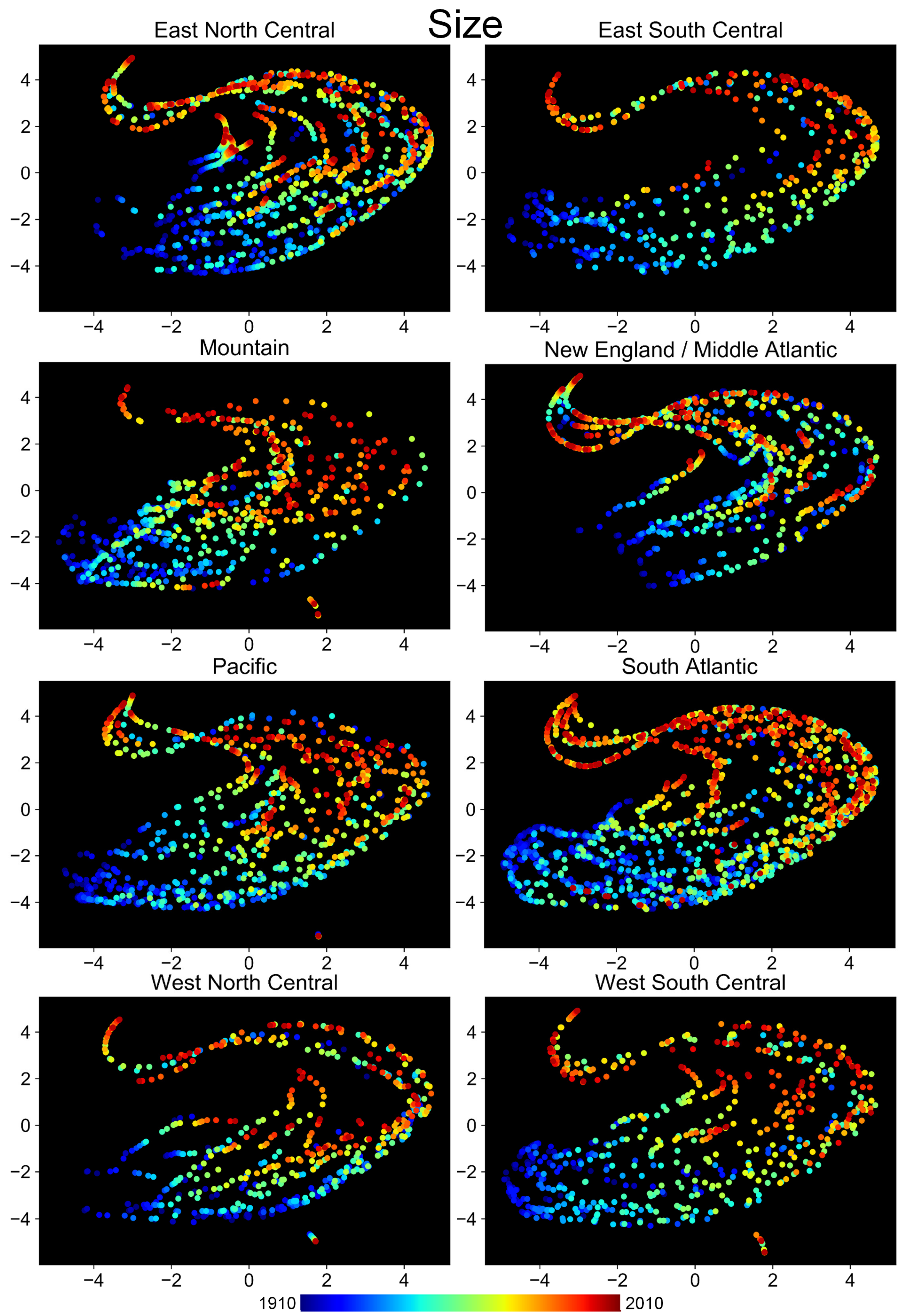

Supplementary Figure 4: Regional decomposition of cross-temporal MSA trajectories for size-related variables in t-SNE[7] space.

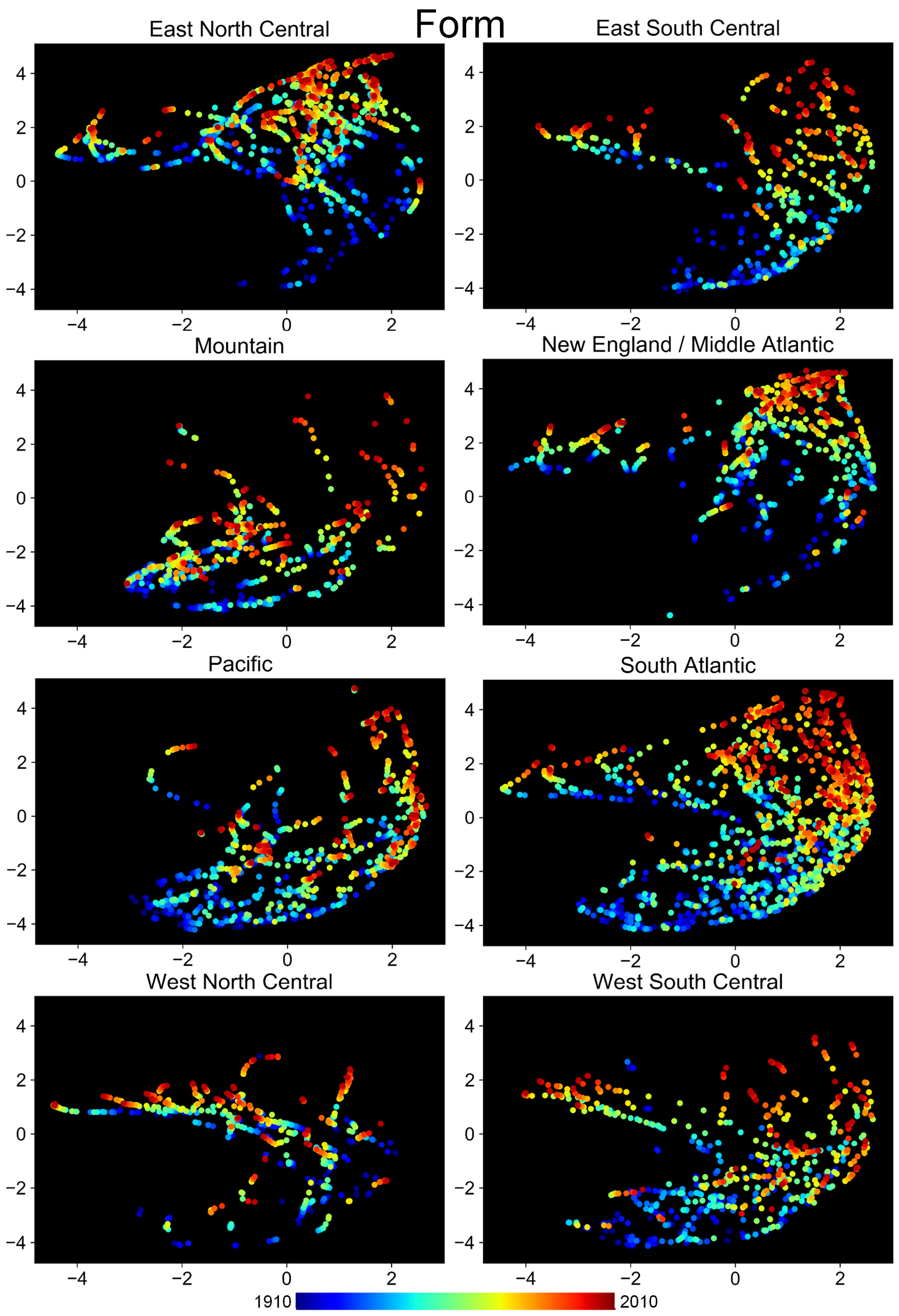

Supplementary Figure 5: Regional decomposition of cross-temporal MSA trajectories for form-related variables in t-SNE[7] space.

Supplementary Table 2: Quantitative assessment of the complexity of cross-temporal MSA trajectories shown in Supplementary Figures 4 and 5, in t-SNE based bi-dimensional similarity spaces, separately for the size and form domain. We report tbe standard deviations of the azimuths of MSA trajectory vectors between the 1910 and 2010 locations in t-SNE space, as a measure of trajectory diversity.

Region	Size	Form	Form - Size
	Std. Dev.	Std. Dev.	Difference
All divisions	81.5240	94.7877	13.2637
East North Central	93.1819	102.5318	9.3500
East South Central	80.0145	113.6992	33.6847
Mountain	71.3611	106.1031	34.7420
New England / Middle Atlantic	81.3955	106.1884	24.7929
Pacific	81.7372	107.0613	25.3241
South Atlantic	91.9853	102.5149	10.5296
West North Central	76.9514	111.5253	34.5739
West South Central	74.5276	108.9739	34.4463

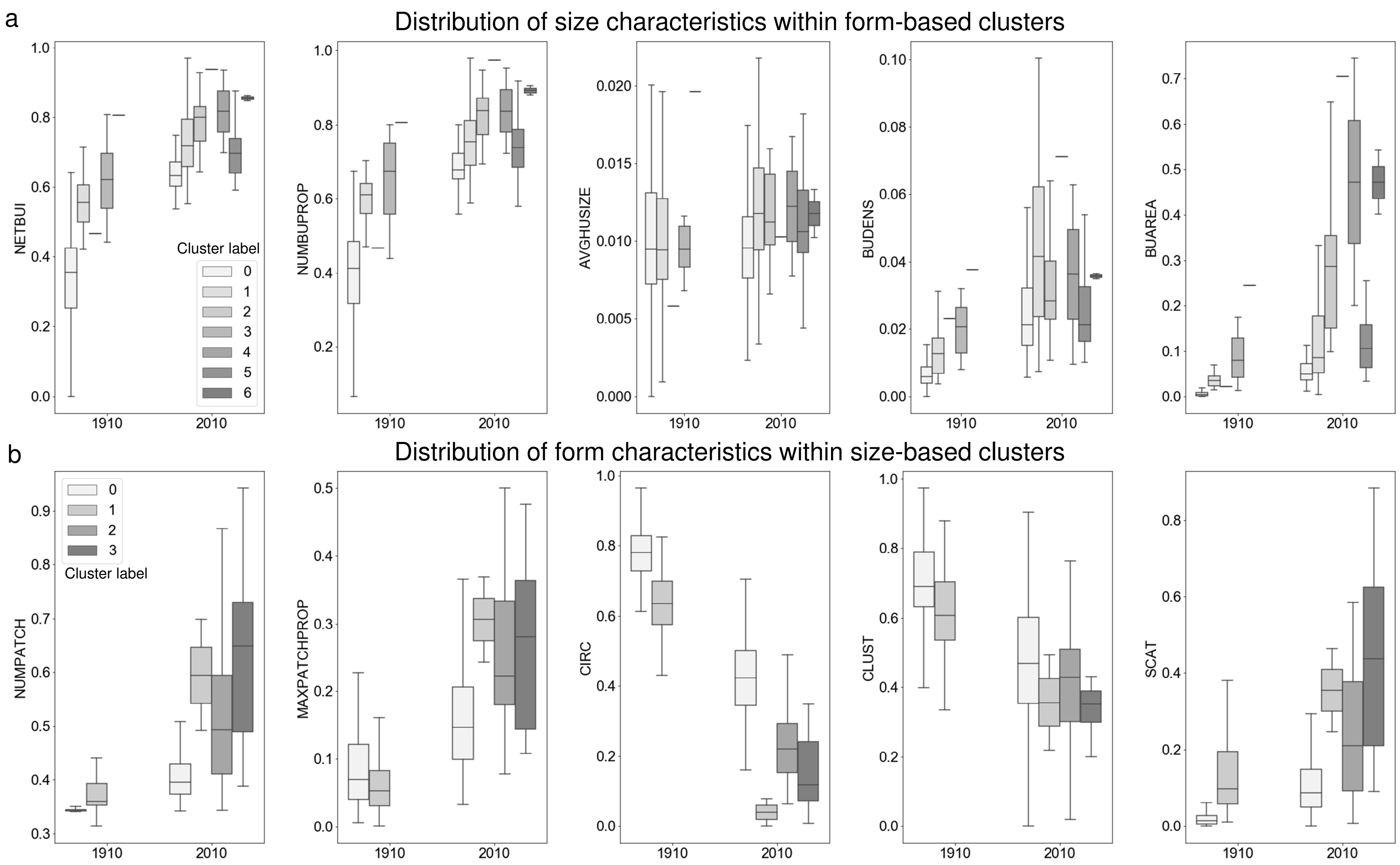

Supplementary Figure 6: Visual assessment of the distributions of size characteristics within form-based MSA clusters, and vice-versa. Data are scaled into range [0,1] for intercomparability purposes. Box-and-whisker plots show medians (horizontal lines), lower and upper quartiles (boxes), and whiskers extend to $1.5 \times IQR$ below the lower quartile, and above the upper quartile, respectively.

Supplementary Table 3: Quantitative assessment of the distributions of size characteristics within form-based MSA clusters, and vice-versa. Median absolute deviations (MAD) are calculated per variable, year and cluster, and the median per variable is reported across all clusters identified in the years 1910 and 2010.

Size variables within form cluster			
Variable	Median MAD in 1910	Median MAD in 2010	Change in %
BUAREA	0,0105	0,0570	441,6383
BUDENS	0,0056	0,0085	51,5028
NETBUI	0,0799	0,0426	-46,7079
AVGHUSIZE	0,0021	0,0021	-1,9754
NUMBUPROP	0,0776	0,0488	-37,1536
**Form variables within size clusters**			
Variable	Median MAD in 1910	Median MAD in 2010	Change in %
MAXPATCHPROP	0,0301	0,0732	142,8904
CIRC	0,0561	0,0637	13,4274
CLUST	0,0802	0,1117	39,2484
NUMPATCH	0,0063	0,0963	1430,4887
SCAT	0,0303	0,1151	279,9403

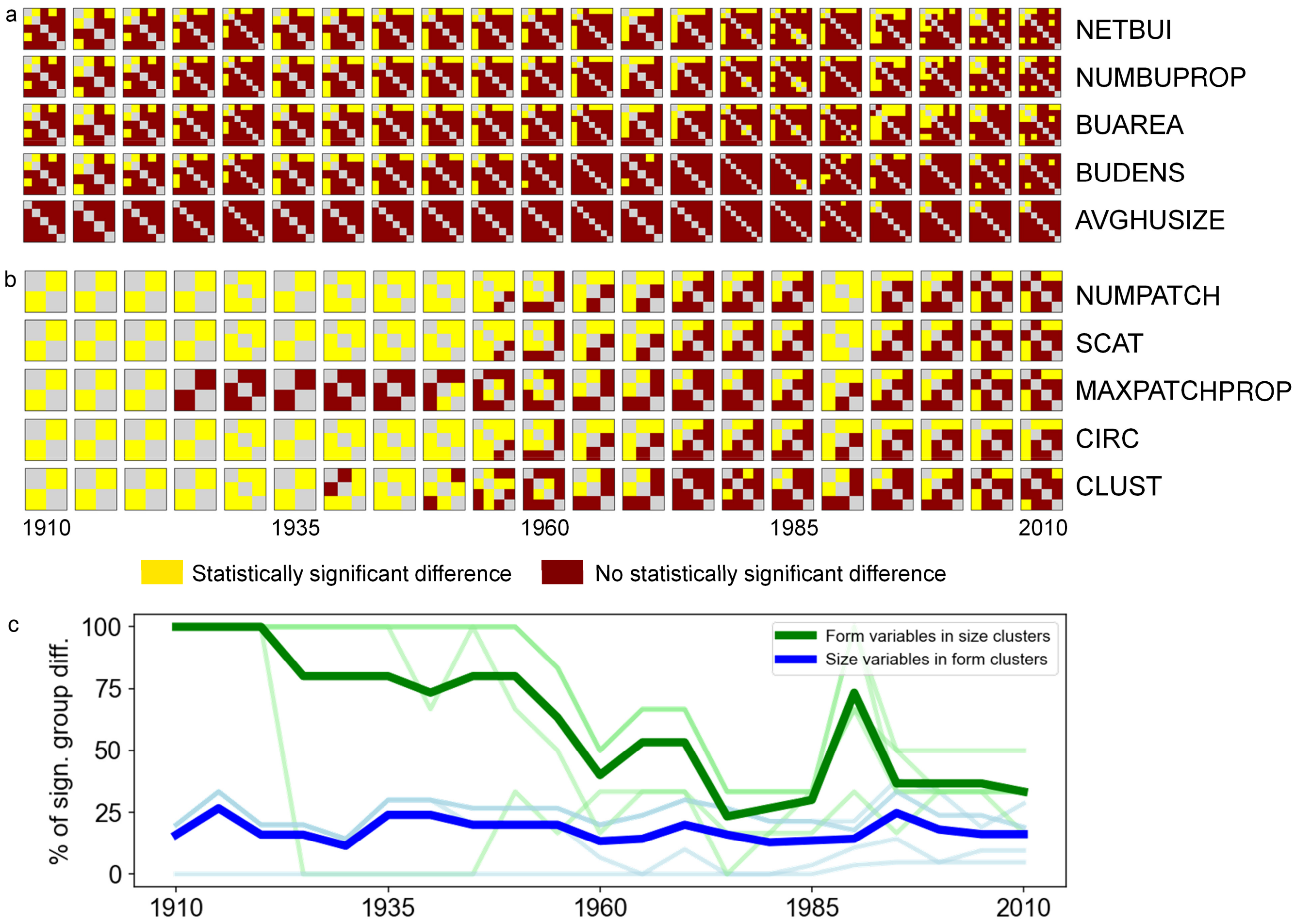

Supplementary Figure 7: Analyzing pairwise differences between (a) size variable distributions within form-based clusters, and (b) form variable distributions within size-based clusters. Each matrix represents the significance / non-significance of Dunn's test of pairwise comparisons[8]. Yellow colors represent p-values <0.05, indicating that the differences between medians of distributions within the respective clusters are statistically significant. (c) shows the percentage of significant differences between clusters per year, averaged across all variables per category. In (c), light line colors represent the percentages per variable, whereas saturated colors represent the respective averages across all size variables (blue) and form variables (green).

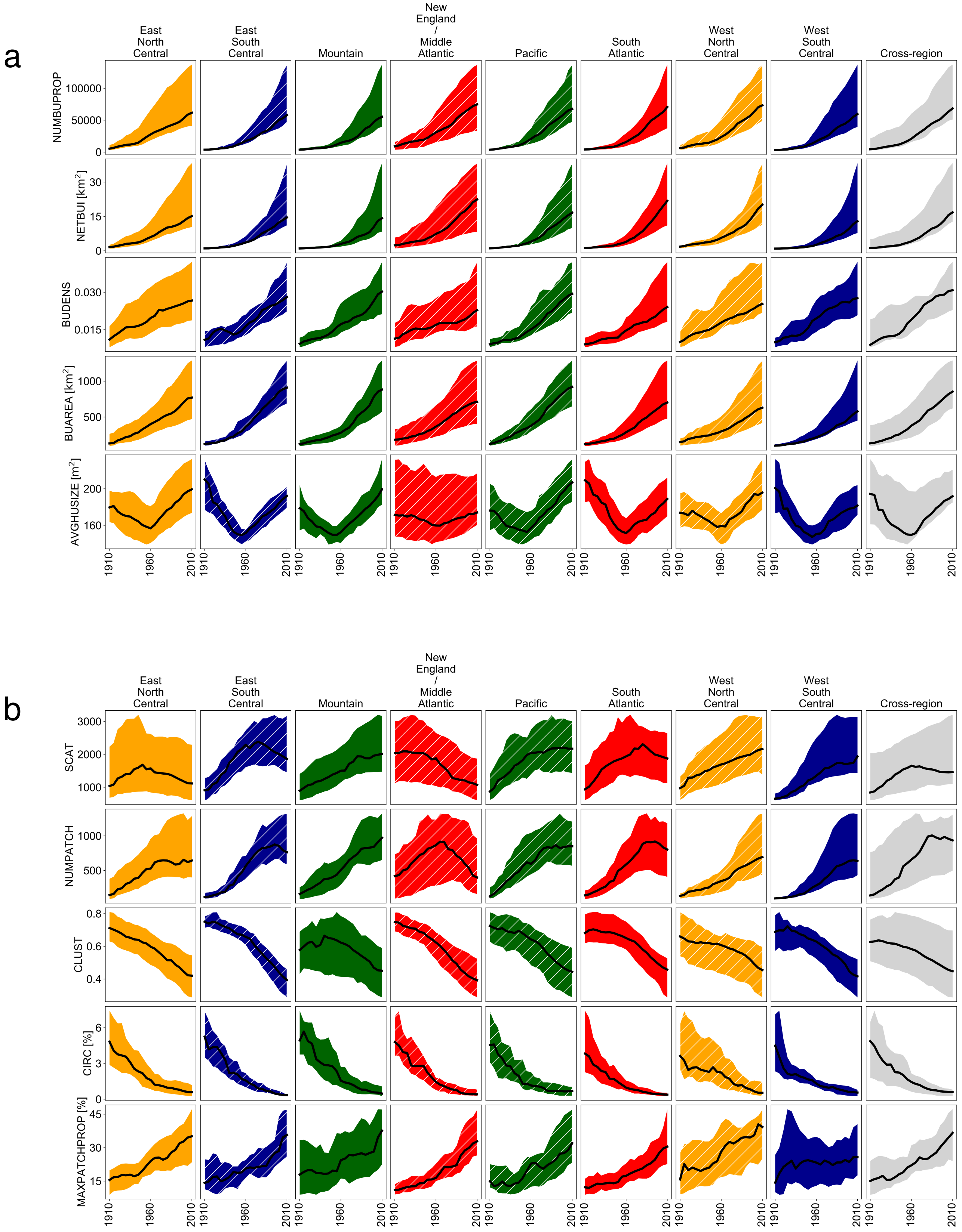

Supplementary Figure 8: Dispersion per variable and region over time: (a) Inter-quartile ranges (color-coded according to main paper Fig. 6a,b,c) and median time series (black lines) per size-density variable, and (b) respective plots for the shape-structure variables. The column to the right shows the cross-region separation, measured by the range of region medians (grey areas) and their median (black lines).

Supplementary Movie 1: MSA bar chart race animation for the NUMPATCH variable (1910 - 2010).

Interactive data animation illustrating the top 15 MSAs per semidecade, ranked by the NUMPATCH variable: `https://public.flourish.studio/visualisation/2765858`

Supplementary Movie 2: MSA bar chart race animation for the NETBUI variable (1910 - 2010).

Interactive data animation illustrating the top 15 MSAs per semidecade, ranked by the NETBUI variable: `https://public.flourish.studio/visualisation/2765807`

Supplementary Movie 3: MSA bar chart race animation for the BUAREA variable (1910 - 2010).

Interactive data animation illustrating the top 15 MSAs per semidecade, ranked by the BUAREA variable: `https://public.flourish.studio/visualisation/2765392`

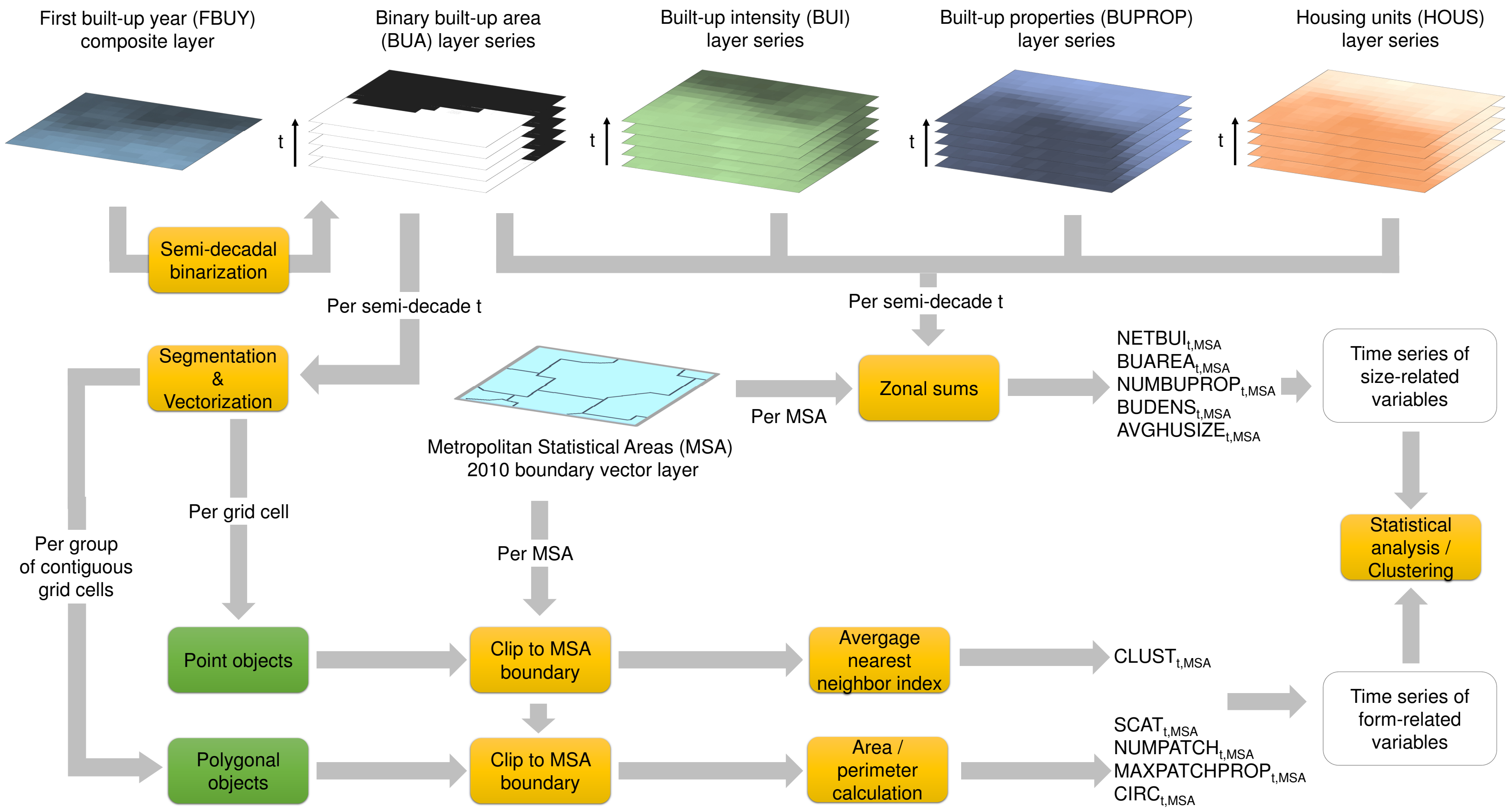

Supplementary Figure 9: Workflow of the performed geospatial data processing and analysis steps implemented in Python. The top row illustrates the datasets contained in the HISDAC-US repository: First built-up year[1], built-up areas[2], built-up intensity[4], built-up properties[3], and housing units[5]. Raster layer processing (i.e., binarization, segmentation), raster-vector operations (i.e., vectorization, clipping, zonal statistics) and average nearest neighbor index[9] calculations were carried out using ESRI ArcGIS Pro 2.5 ArcPy Python package[10]. The time series data (i.e., urban spatial metrics per MSA and semidecade)[11] were generated and processed using Pandas Python package[12]. Data analysis included t-distributed neighbor transform (t-SNE[7]), Principal Component Analysis (PCA)[13], BIRCH ("Balanced Iterative Reducing and Clustering using Hierarchies") cluster analysis[14,15], and was carried out using Scikit-learn Python package[16]. Statistical tests including Kruskal-Wallis tests[17], Dunn's test of pairwise comparisons[8], and Shapiro-Wilk normality tests[6] were carried out using SciPy Python package[18]. Other analyses were performed using the NumPy Python package[19]. Data visualization was carried out using Matplotlib[20] and Seaborn[21] Python packages, as well as ESRI ArcMap[22], and data animations were done using the Flourish data visualization application[23].

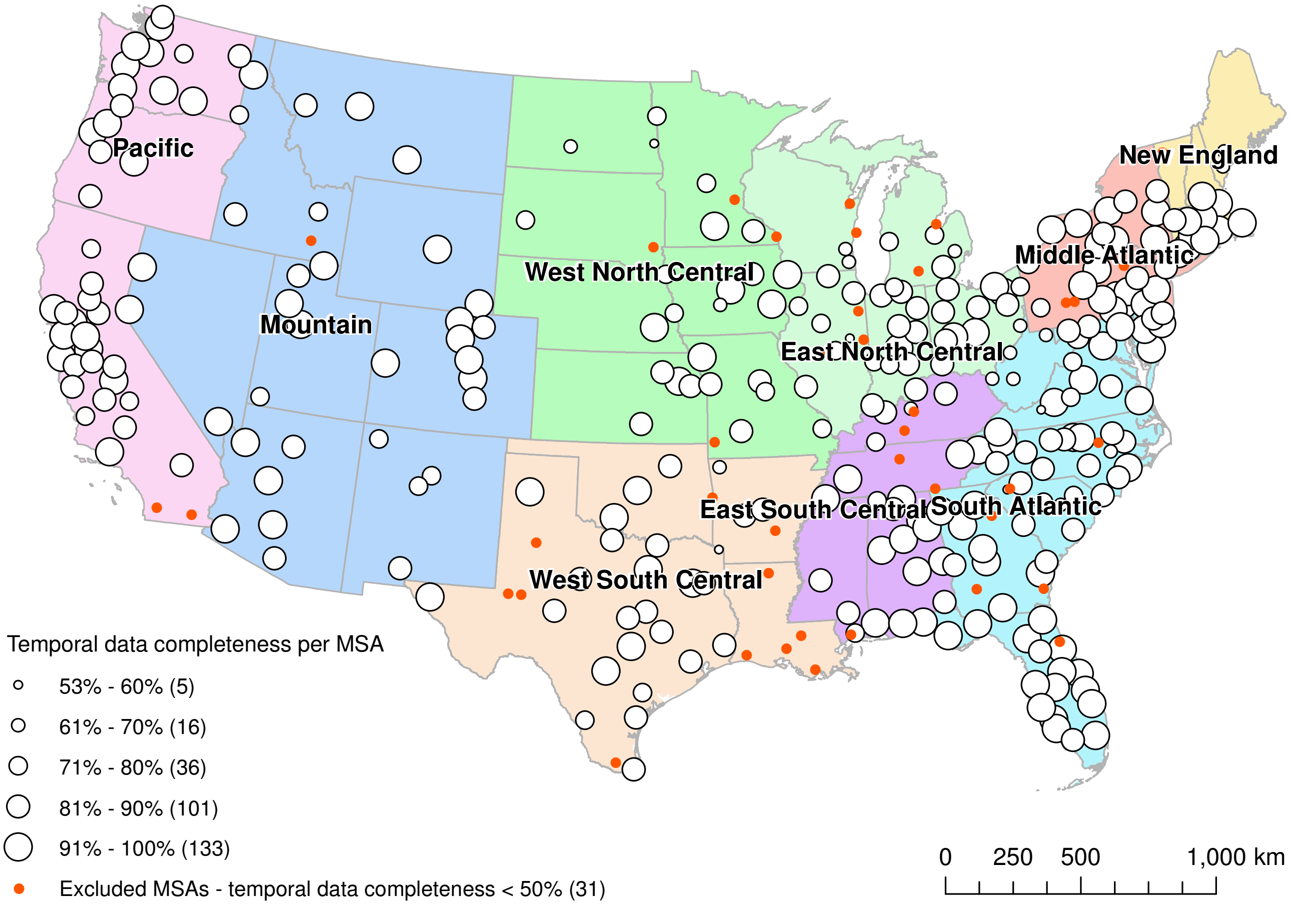

Supplementary Figure 10: Temporal attribute completeness map in the used HISDAC-US data per MSA. Frequencies per completeness class are shown in parentheses. MSA locations represent centroids derived from MSA boundaries obtained from US Census Bureau[24], also shown are US states and census division boundaries obtained from US Census Bureau[25,26].

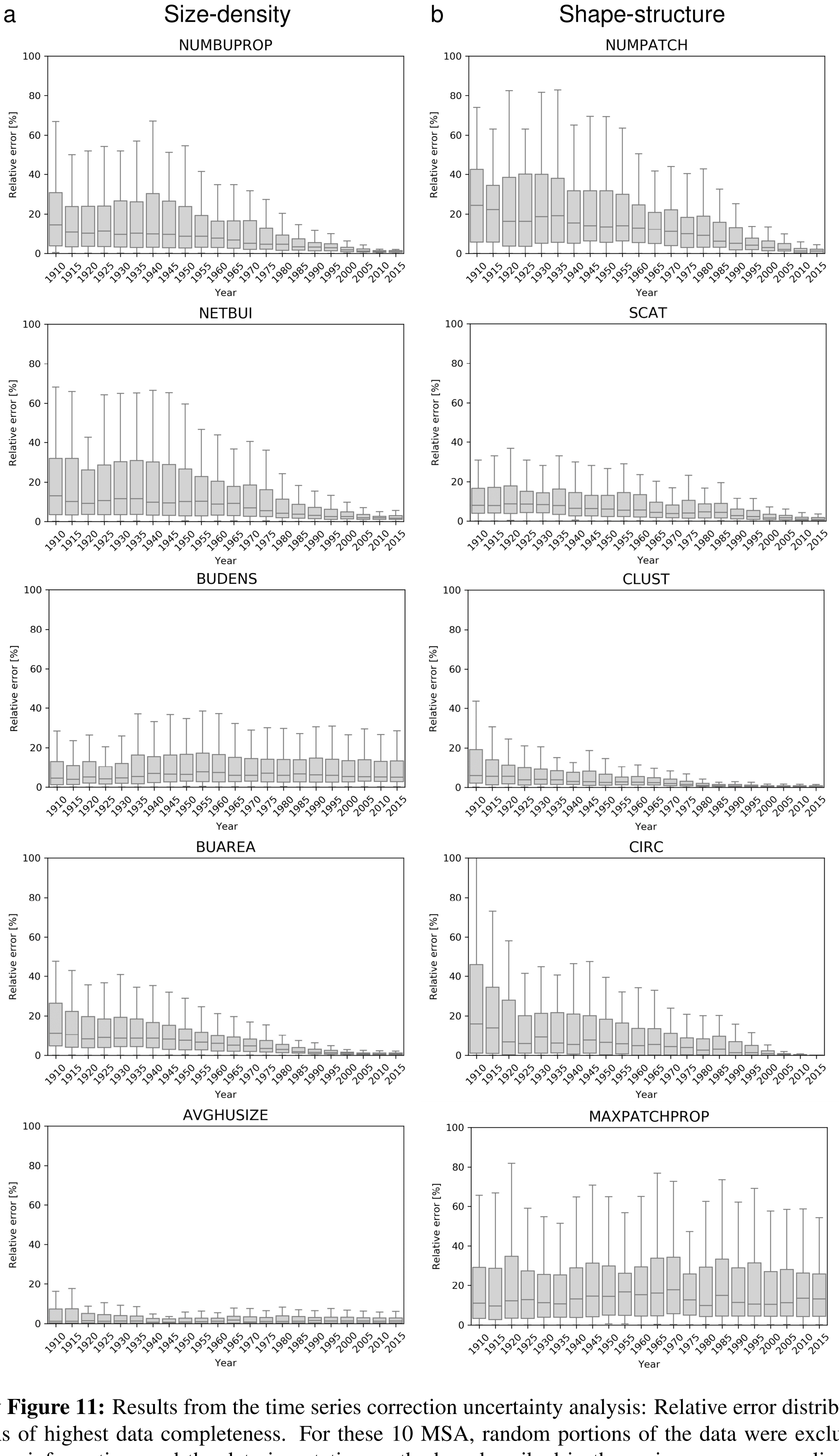

Supplementary Figure 11: Results from the time series correction uncertainty analysis: Relative error distributions over time for the 10 MSAs of highest data completeness. For these 10 MSA, random portions of the data were excluded, simulating missing built year information, and the data imputation method as described in the main paper was applied to account for these data gaps. Based on the imputed values and the actual values, relative error distributions were obtained for each year. Results indicate increasing, but acceptable levels of uncertainty in earlier years for most variables. Box-and-whisker plots show medians (horizontal lines), lower and upper quartiles (boxes), and whiskers extend to $1.5 \times IQR$ below the lower quartile, and above the upper quartile, respectively.

Supplementary References

[1] Leyk, S. & Uhl, J. H. Historical settlement composite layer for the U.S. 1810 - 2015 (2018). URL https://doi.org/10.7910/DVN/PKJ90M.

[2] Uhl, J. H. & Leyk, S. Historical built-up areas (BUA) - gridded surfaces for the U.S. from 1810 to 2015 (2020). URL https://doi.org/10.7910/DVN/J6CYUJ.

[3] Uhl, J. H. & Leyk, S. Historical built-up property locations (BUPL) - gridded surfaces for the U.S. from 1810 to 2015 (2020). URL https://doi.org/10.7910/DVN/SJ213V.

[4] Leyk, S. & Uhl, J. H. Historical built-up intensity layer series for the U.S. 1810 - 2015 (2018). URL https://doi.org/10.7910/DVN/1WB9E4.

[5] Uhl, J. H. & Leyk, S. Historical built-up property records (BUPR) - gridded surfaces for the U.S. from 1810 to 2015 (2020). URL https://doi.org/10.7910/DVN/YSWMDR.

[6] Shapiro, S. S. & Wilk, M. B. An analysis of variance test for normality (complete samples). *Biometrika* **52**, 591–611 (1965).

[7] van der Maaten, L. & Hinton, G. Visualizing data using t-SNE. *J. Mach. Learn. Res.* **9**, 2579–2605 (2008).

[8] Dunn, O. J. Multiple comparisons using rank sums. *Technometrics* **6**, 241–252 (1964).

[9] Clark, P. J. & Evans, F. C. Distance to nearest neighbor as a measure of spatial relationships in populations. *Ecology* **35**, 445–453 (1954).

[10] ESRI. ArcPy. https://pro.arcgis.com/en/pro-app/arcpy/ (2019).

[11] Uhl, J. H., Connor, D. S., Leyk, S. & Braswell, A. E. Urban spatial metrics for metropolitan statistical areas in the u.s. from 1910 to 2010 (2020). URL https://figshare.com/articles/dataset/Urban_spatial_metrics_for_Metropolitan_Statistical_Areas_in_the_U_S_from_1910_to_2010/13303091/1.

[12] The pandas development team. pandas-dev/pandas: Pandas. https://doi.org/10.5281/zenodo.3509134 (2019).

[13] Wold, S., Esbensen, K. & Geladi, P. Principal component analysis. *Chemom. Intell. Lab. Syst.* **2**, 37–52 (1987).

[14] Zhang, T., Ramakrishnan, R. & Livny, M. BIRCH: an efficient data clustering method for very large databases. In *ACM Sigmod Rec.*, vol. 25, 103–114 (ACM, 1996).

[15] Lorbeer, B. *et al.* Variations on the clustering algorithm BIRCH. *Big data Res.* **11**, 44–53 (2018).

[16] Buitinck, L. *et al.* API design for machine learning software: experiences from the scikit-learn project. In *ECML PKDD Workshop: Languages for Data Mining and Machine Learning*, 108–122 (2013).

[17] Kruskal, W. H. & Wallis, W. A. Use of ranks in one-criterion variance analysis. *J. Am. Stat. Assoc.* **47**, 583–621 (1952).

[18] Virtanen, P. *et al.* SciPy 1.0: Fundamental Algorithms for Scientific Computing in Python. *Nature Methods* (2020).

[19] Oliphant, T. E. *A guide to NumPy*, vol. 1 (Trelgol Publishing USA, 2006).

[20] Hunter, J. D. Matplotlib: A 2d graphics environment. *Computing in Science & Engineering* **9**, 90–95 (2007).

[21] Waskom, M. *et al.* mwaskom/seaborn: v0.8.1 (september 2017) (2017). URL https://doi.org/10.5281/zenodo.883859.

[22] ESRI. ArcMap. https://desktop.arcgis.com/en/arcmap/ (2019).

[23] Kiln Enterprises Ltd. Flourish data visualization and storytelling. https://flourish.studio/ (2019).

[24] US Census Bureau. Core-based statistical areas 2010. https://www2.census.gov/geo/tiger/TIGER2010/CBSA/2010/ (2018). Online; accessed 01 January 2020.

[25] US Census Bureau. TIGER/Line Shapefiles: US state boundary file 2010. https://www2.census.gov/geo/tiger/TIGER2010/STATE/2010/tl_2010_us_state10.zip (2011). Online; accessed 25 November 2020.

[26] US Census Bureau. 2014 Census Bureau Region and Division Codes and State FIPS Codes. https://www.census.gov/geographies/reference-files/2014/demo/popest/2014-geocodes-all.html (2014). Online; accessed 01 January 2020.
